# eVIP2: Expression-based variant impact phenotyping to predict the function of gene variants

**DOI:** 10.1371/journal.pcbi.1009132

**Published:** 2021-07-02

**Authors:** Alexis M. Thornton, Lishan Fang, April Lo, Maria McSharry, David Haan, Casey O’Brien, Alice H. Berger, Marios Giannakis, Angela N. Brooks

**Affiliations:** 1 Department of Biomolecular Engineering, University of California Santa Cruz, Santa Cruz, California, United States of America; 2 UCSC Genomics Institute, University of California Santa Cruz, Santa Cruz, California, United States of America; 3 Department of Medical Oncology, Dana-Farber Cancer Institute, Harvard Medical School, Boston, Massachusetts, United States of America; 4 Broad Institute of Massachusetts Institute of Technology and Harvard, Cambridge, Massachusetts, United States of America; 5 Department of Orthopedics, The Eight Affiliated Hospital of Sun Yat-sen University, Shenzhen, China; 6 Fred Hutchinson Cancer Research Center, Seattle, Washington, United States of America; Johns Hopkins University, UNITED STATES

## Abstract

While advancements in genome sequencing have identified millions of somatic mutations in cancer, their functional impact is poorly understood. We previously developed the expression-based variant impact phenotyping (eVIP) method to use gene expression data to characterize the function of gene variants. The eVIP method uses a decision tree-based algorithm to predict the functional impact of somatic variants by comparing gene expression signatures induced by introduction of wild-type (WT) versus mutant cDNAs in cell lines. The method distinguishes between variants that are gain-of-function, loss-of-function, change-of-function, or neutral. We present eVIP2, software that allows for pathway analysis (eVIP Pathways) and usage with RNA-seq data. To demonstrate the eVIP2 software and approach, we characterized two recurrent frameshift variants in RNF43, a negative regulator of Wnt signaling, frequently mutated in colorectal, gastric, and endometrial cancer. RNF43 WT, RNF43 R117fs, RNF43 G659fs, or GFP control cDNA were overexpressed in HEK293T cells. Analysis with eVIP2 predicted that the frameshift at position 117 was a loss-of-function mutation, as expected. The second frameshift at position 659 has been previously described as a passenger mutation that maintains the RNF43 WT function as a negative regulator of Wnt. Surprisingly, eVIP2 predicted G659fs to be a change-of-function mutation. Additional eVIP Pathways analysis of RNF43 G659fs predicted 10 pathways to be significantly altered, including TNF-α via NFκB signaling, KRAS signaling, and hypoxia, highlighting the benefit of a more comprehensive approach when determining the impact of gene variant function. To validate these predictions, we performed reporter assays and found that each pathway activated by expression of RNF43 G659fs, but not expression of RNF43 WT, was identified as impacted by eVIP2, supporting that RNF43 G659fs is a change-of-function mutation and its effect on the identified pathways. Pathway activation was further validated by Western blot analysis. Lastly, we show primary colon adenocarcinoma patient samples with R117fs and G659fs variants have transcriptional profiles similar to BRAF missense mutations with activated RAS/MAPK signaling, consistent with KRAS signaling pathways being GOF in both variants. The eVIP2 method is an important step towards overcoming the current challenge of variant interpretation in the implementation of precision medicine. eVIP2 is available at https://github.com/BrooksLabUCSC/eVIP2.

## Author summary

Cancer is often caused by a DNA change, called a mutation, that creates an atypical protein variant. Many cancer-associated mutations remain uninvestigated because experimental validation of their effect on gene function is costly, time-consuming, and requires prior knowledge of a gene’s function. We present our computational tool, eVIP2, which advances our previous approach called expression-based variant impact phenotyping (eVIP) and can be used with RNA sequencing data. This method uses gene expression data to characterize a gene variant’s function, requiring no prior knowledge of the wild-type gene’s function. With eVIP2, we can predict if a mutation causes a gain, loss, or change in function, or if it is neutral. Here, we found two recurrent frameshift mutations in *RNF43* that have different effects on gene function where one mutation causes multiple cancer pathways to be activated. This study highlights the usefulness of using the eVIP2 tool for profiling a mutation’s impact at the pathway level.

This is a *PLOS Computational Biology* Software paper.

## Introduction

While advancements in genome sequencing have identified millions of somatic mutations in cancer [1–4], interpretation of these variants remains a major challenge to the implementation of precision medicine. Distinct assays are generally used to determine mutation impact for each individual gene being studied, slowing down the process of variant interpretation.

Previously, no single assay could rapidly profile the functional impact of a diverse set of genes. Earlier studies demonstrated the feasibility of using gene expression signatures as “fingerprints” of molecular function [5]. Kim et al. characterized the function of rare variants by illustrating they can have similar gene expression signatures to variants with known functions [6].

In Berger et al., we presented the expression-based variant-impact phenotyping (eVIP) method that uses gene expression changes to distinguish impactful from neutral somatic mutations [7]. This study used the L1000 Luminex bead-based gene expression assay, which measures the abundance of 978 “landmark” genes [8]. eVIP was used to characterize 194 somatic mutations in 53 genes identified in primary lung adenocarcinomas, demonstrating the feasibility of systematic functional interpretation of variants using gene expression data.

Here, we present advancements in the eVIP approach and software, called eVIP2. eVIP2 includes a more automated pipeline and improvements in user-friendliness. Where eVIP used the L1000 assay, eVIP2 allows for input of RNA-seq data, which is more widely used and provides for a more complete profile of the transcriptome. Using RNA-seq also gives the ability to do pathway analysis. In addition to functional predictions on the overall effect of each variant, we also include an approach called eVIP Pathways to predict the functional impact on specific cellular signaling pathways. We show that eVIP2 can be used with RNA-seq to characterize variants of unknown function and identify their downstream effect on cellular signaling pathways.

## Design and implementation

Upon overexpression of wild-type (WT) and mutant alleles, eVIP compares transcriptome changes to predict the mutant’s impact as neutral, loss-of-function (LOF), gain-of-function (GOF), or change-of-function (COF) compared to the WT (Table 1). Overexpression of a neutral variant has the same effect as the overexpression of the WT. Overexpression of a loss-of-function (LOF) allele causes no effect or a mild effect when compared to the overexpression of the WT allele. Overexpression of a gain-of-function (GOF) allele causes a strong effect when compared to the WT. Lastly, a change-of-function (COF) allele has a different effect compared to WT but a LOF or GOF call does not reach statistical significance.

**Table 1:** Transcriptome-centric definitions of eVIP mutation impact calls.

eVIP call	Effect on gene expression changes of mutant ORF overexpression relative to WT ORF overexpression
Neutral	Same as WT
Loss-of-function (LOF)	No effect or mild effect compared to WT
Gain-of-function (GOF)	Strong effect compared to WT
Change-of-function (COF)	Different effect compared to WT but LOF or GOF call does not reach statistical significance

### Algorithm for expression-based variant impact phenotyping

**Fig 1:**
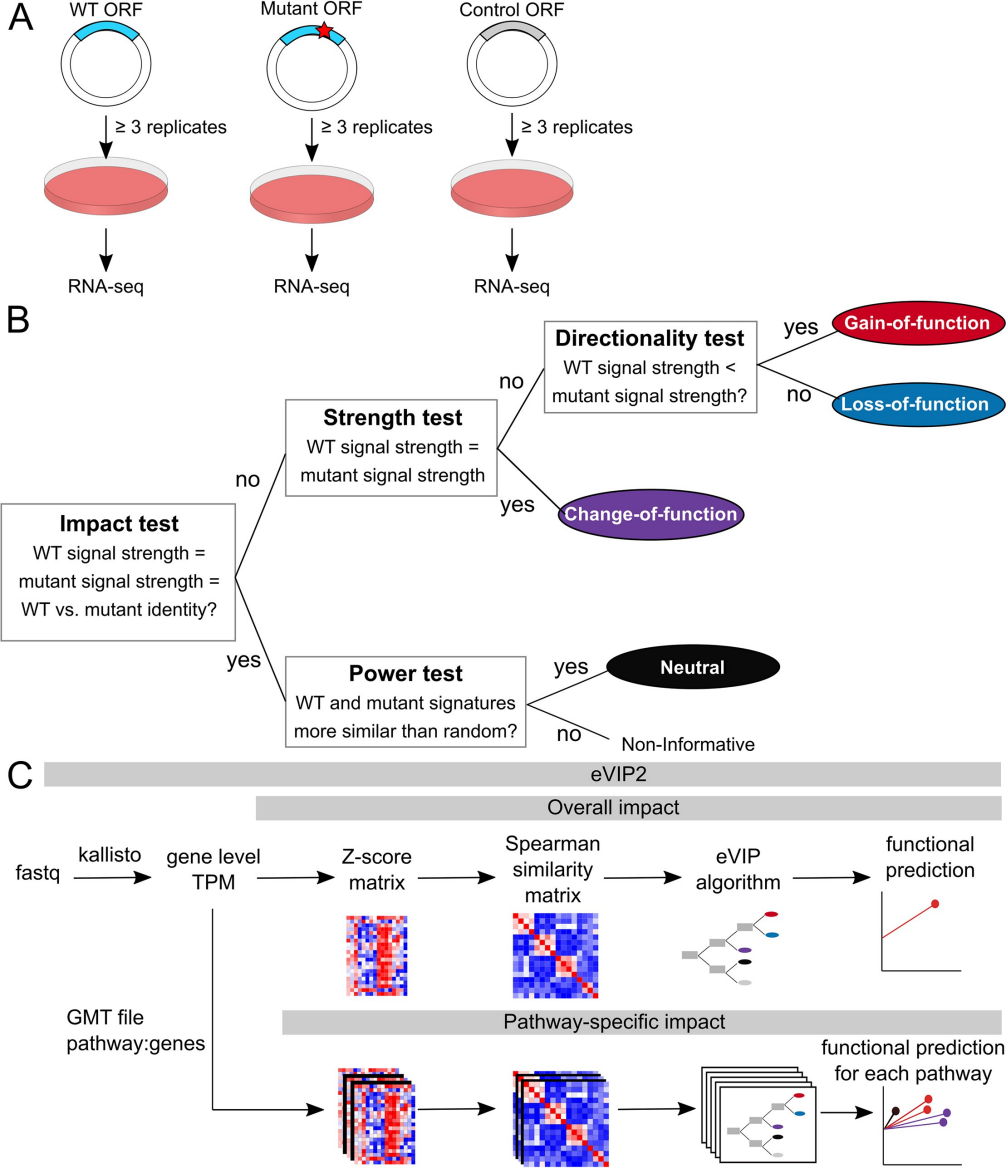
The eVIP algorithm uses RNA-seq data to predict the function of somatic mutations. (A) Overview of the experimental approach (B) Schematic of the eVIP decision tree-based eVIP algorithm. The impact test is a Kruskal-Wallis test of the three distributions: wild-type replicate self-correlation, mutant replicate self-correlation, and wild-type versus mutant correlation. It outputs a Bonferonni-adjusted p-value, which represents the likelihood of mutation impact. Impactful mutations are then tested for their directional impact of gain-of-function (GOF) or loss-of-function (LOF). For non-impactful mutations, a “power test” determines whether the two signatures are similar to one another due to a real signal or due to noise. If they are similar, the mutation is considered to have a neutral impact [7]. (C) Overview of the eVIP2 pipeline which incorporates overall impact and pathway impact. The eVIP2 pipeline uses gene level counts (TPM) to predict the functional impact of a mutation. Many mutations can be processed in parallel. There is the option for eVIP Pathways, which predicts the impact of each mutation on a pathway.

The eVIP approach assumes that the underlying expression data were derived from cell line experiments from overexpression of a WT ORF, mutant ORF(s), and control ORF(s) [7](Fig 1A). The eVIP method uses a decision tree-based algorithm that determines the functional impact of a mutation by comparing gene expression signatures induced by wild-type and mutant ORFs (Fig 1B)[7]. The two features of gene expression changes that eVIP uses are signal strength and signature identity. The signal strength is a quantitative measure of replicate consistency (WT vs. WT or variant vs. variant)[7]. The pairwise replicate correlations for a given allele’s signature is calculated using Spearman rank correlation. A strong signal among a WT or a variant’s replicates indicates a strong expression signature that is internally consistent and has a high signal-to-noise ratio. A weak signal is inconsistent from replicate to replicate.

The signature identity is found by calculating the correlation between the transcripts in the wild-type signature and those in the mutant signature (WT vs. variant)[7]. Mutant ORFs that function similarly to WT ORFs induce similar gene expression changes, giving highly correlated gene expression signatures. The correlation is calculated between each replicate signature of a mutant ORF with the replicate of its WT ORF.

At the root of the decision tree, we test the null hypothesis that the mutant signature and the wild-type signature are indistinguishable, and give the overall impact prediction p-value for the given variant[7]. This is based on a Kruskal-Wallis test performed on three distributions: wild-type replicate self-correlation, mutant replicate self-correlation, and wild-type versus mutant correlation. Kruskal-Wallis is nonparametric and is used to determine if there are significant differences between the three distributions. Benjamini-Hochberg False Discovery Rate correction is applied and when the null hypothesis is rejected, the variant is impactful. The variant can then be further characterized as a change-of-function (COF), gain-of-function (GOF), or loss-of-function (LOF). A non-parametric two-sided Wilcoxon test is done to test the null hypothesis that there is no difference in self-replicate correlation between the mutant and the wild type. If the difference is not significant the variant is a change-of-function.

If the difference is significant, we then look to find the direction of the difference in self-replicate correlation. When the mutant self-replicate correlation is less than wild-type self-replicate correlation, it has a negative impact score and is considered LOF. As mentioned above, self-replicate correlation of an LOF variant that is less than the WT suggests the variant has no effect or mild effect compared to WT. When it is greater, it has a positive impact score, and the variant is a GOF.

When the overall impact p-value is greater than the threshold, the variant may have no impact on gene function (neutral) or be non-informative. A non-parametric two-sided Wilcoxon test is then done by comparing the wild-type versus mutant replicate correlation distribution to a null distribution. The null distribution is determined by comparing each mutant signature to each control signature. Thus, a variant is predicted to be neutral if the mutant signature is indistinguishable from wild-type and if the mutant signature is more similar to wild-type than control signature comparisons. If the WT and/or mutant signatures are noisy and not indistinguishable from the null distribution, it will be classified as non-informative.

### Data processing and eVIP2 pipeline

The new version of eVIP, eVIP2, improves usability by combining multiple steps into a single python command and adds eVIP Pathway functionality. For overall functional predictions, the eVIP2 software takes in L1000 Z-scores or RNA-seq gene level transcript per million (TPM) counts (Fig 1C). When using RNA-seq, low expressed genes (TPM < 1) are removed and the data is log2 transformed.

For pathway analysis, the tximport package in R is used to import transcript level counts and convert to gene level expression[9]. We use Kallisto for quantification, but gene expression tables generated from other tools can also be used [10].

From the log2 TPM counts, eVIP2 transforms the values to Z-scores using the mean and standard deviation across the replicates in all conditions, including the controls. The data is then processed to a sample-by-sample self-correlation matrix. It is recommended to have at least three biological replicates for each condition; however, more replicates will allow for increased statistical power[11]. In the original implementation using L1000 data, a weighted connectivity score was used as a measure of correlation. Using the large L1000 dataset that tested the impact of 194 somatic mutations [7], we compared the weighted connectivity score to Spearman rank as a measure of correlation. We found that the corrected p-values from the impact test using either correlation method were comparable (S1 Fig); therefore, we used Spearman rank correlation for RNA-Seq data. Using the correlation matrix, the described eVIP algorithm is run to give a prediction of LOF, GOF, COF, Neutral, or non-informative.

In addition to a variant receiving an overall functional impact prediction, with eVIP Pathways, the user can also determine pathway-specific functional calls, which allows for more specific functional analysis. eVIP Pathways uses a Gene Matrix Transposed (GMT) file representing the mapping of pathways to their genes. Custom gene sets or curated gene sets from MsigDB, Kegg, and Reactome can be used [12–14].

eVIP Pathway analysis differs from existing pathway tools like GSEA that perform enrichment analysis. For pathway analysis, eVIP Pathways first finds differentially expressed genes that are specific to the WT or mutant. The WT gene and each mutant are compared to the control using DESeq2 [15]. We define mutation-specific genes as genes that are differentially expressed only in the control vs mutation and not in the control versus WT. These genes represent a new function caused by the mutant. The WT-specific genes are differentially expressed only in the control versus WT and not in the control versus mutant. These are genes that are expected to be affected by normal WT function but are not affected by the mutant, and therefore represent mutant loss of function. eVIP Pathways is then run separately using the WT-specific and mutant-specific genes (with multiple-testing correction).

A standard approach to understanding the function of a mutation is to look at differentially expressed genes in cells with and without induced expression of the mutant [16–19]. We believe that analysis of mutant-specific differentially expressed genes allows a better discernment of mutation function, by disregarding the preserved effects of the WT gene.

## Results

### eVIP results are consistent between L1000 and RNA-seq

As the eVIP method was originally applied on the L1000 weighted connectivity score, we aimed to determine if eVIP could also be used with Spearman rank correlation on RNA-seq data. To compare the eVIP approach with L1000 data versus with RNA-seq data, we investigated ARAF variants that were analyzed previously [7]. With L1000, ARAF p.V145L was determined to be neutral and ARAF p.D429A, ARAF p.S214C, ARAF p.S214F, were impactful variants and these variant function predictions were consistent with their effect on xenograft tumor formation and an erlotinib-rescue assay[7]. Given that ARAF had validated neutral and impactful variants, we selected these to test our RNA-Seq approach, in the same A549 cell lines. For the RNA-seq experiment, we selected the ARAF p.V145L neutral variant and one of the impactful variants p.S214F to test. The eVIP functional impact prediction of these two variants was identical when using RNA-seq (S2 Fig and S1 and S2 Files).

An additional two variants, ARAF p.S214C.p.D429A and ARAF p.S214F.p.D429A, were evaluated with eVIP2 that were not analyzed with eVIP in the original study due to quality control filtering. These variants are double mutants which introduces an additional kinase inactivating p.D429A mutation. Our mouse xenograft assay and erlotinib-rescue assay showed that the additional p.D429A mutation reverts the GOF activity of S214C and S214F mutants [7]. eVIP2 determined both double mutants to be neutral (S2 Fig). The calls did not change when using RNA-seq with only genes measured in the L1000 assay (S2 Fig and S2 File).

### False discovery rate estimation

Eight replicates were used with the L1000 eVIP approach, however due to cost, the number of replicates was reduced to four for the RNA-seq experiments. We chose to use four replicates instead of the standard three to have more statistical power. To adjust for less statistical power compared with eight replicates, we adjusted the eVIP algorithm thresholds from 0.05 to 0.1. To evaluate the false discovery rate (FDR) using RNA-Seq data with the eVIP approach, we created mock comparisons using 12 replicates from an independent empty vector RNA-seq experiment in A549 cells. For 1000 iterations, we chose a random 4 replicates to represent a mock mutant and a different set of 4 random replicates to represent a mock WT. Therefore, we expect eVIP to determine the mock mutant to be “neutral” and any calls of LOF, COF, or GOF were considered a false positive. The FDR rate was 2.8%, 0.304%, and 0.406% for the overall eVIP2 calls, the WT-specific pathway calls, and the mutation-specific pathway calls respectively, suggesting the eVIP FDR cut-off of 10% is well-calibrated.

### eVIP2 identifies different impacts of two frameshift mutations in *RNF43*

**Fig 2:**
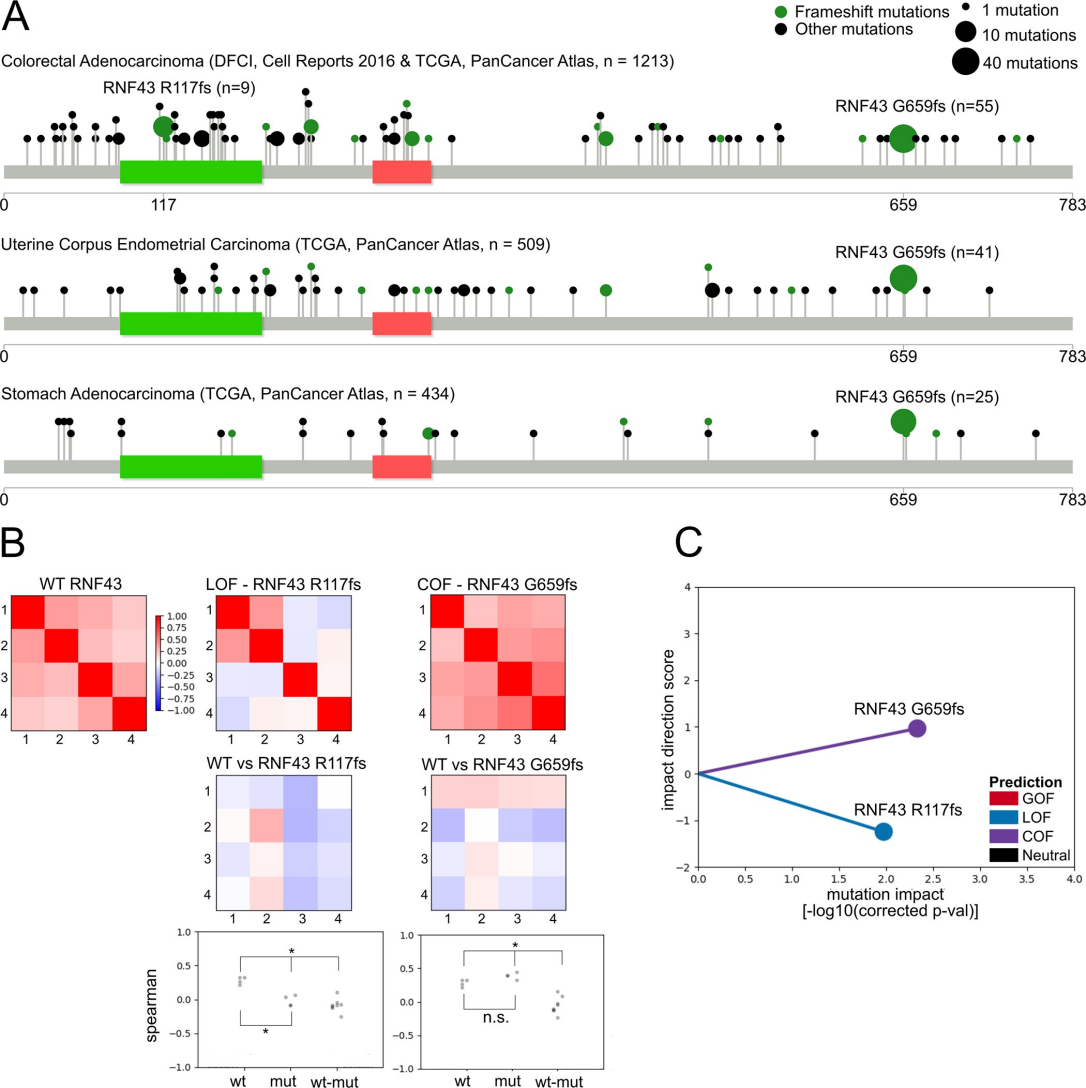
eVIP identifies functional differences between two RNF43 frameshift mutations. (A) Lollipop plots [20] showing the frequency of mutations in RNF43 in three different cohorts[21,22]. The ZNRF-3 ectodomain is indicated in green and the ring finger domain is indicated in red. (B) Heat map representation of WT replicate consistency (WT vs WT) or variant replicate consistency (RNF43 G659fs vs RNF43 G659fs and RNF43 R117fs vs RNF43 R117fs). Values correspond to Spearman rank correlation. Signature identity (WT vs variant) is represented by heatmaps in the second row. Dot-plot representation of replicate consistency and signature identity measured by Spearman rank correlation. *, adjusted p < 0.1. n.s., adjusted p > 0.1. (C) A “sparkler” plot representation of eVIP predictions[7]. A point represents a variant. The x-axis represents the Kruskal Wallis “impact test” -log10(adjusted p-value). The y-axis is the “impact direction score”, the absolute value of which is equal to the–log10 (adjusted p-value) of a Wilcoxon test directly comparing wild-type and mutant ORF replicate consistency. The sign of the impact direction score is positive if the mutant replicate consistency is greater than WT and negative if the mutant replicate consistency is less than the WT replicate consistency.

To demonstrate the utility of the eVIP2 approach, we examined the impact on *RNF43* gene function of its most common mutations, R117fs and G659fs (Fig 2A). *RNF43* encodes for a cell-surface transmembrane E3 ubiquitin-protein ligase that acts as a negative regulator of the Wnt signaling pathway [23–25]. Over 18% of colorectal adenocarcinomas and endometrial carcinomas have *RNF43* mutations [26] with p.G659Vfs*41 and p.R117Afs*41 being the most common. *RNF43* mutations are associated with microsatellite-instable tumors and are mutually exclusive with inactivating APC mutations [26]. Recurrent *RNF43* mutations are predicted to create neopeptides [22,27] and have been associated with aggressive tumor biology [28]. The high frequency of the *RNF43 G659fs* variant suggested it could have a different functional impact than other truncating variants. Moreover, despite being a hotspot mutation, a previous study showed that *RNF43 G659fs* is a passenger mutation because it maintains negative regulation of the Wnt pathway [29]; therefore, we believed the RNF43 frameshift variants would be an interesting use case for eVIP2.

Wild-type *RNF43* and both frameshift mutations were overexpressed in quadruplicate in HEK293T cells, and expression profiling was performed using RNA-Seq. HEK293T cells have the advantage of having low levels of endogenous *RNF43*, which simplifies interpretation of the result of introducing our overexpression constructs. The overexpression of *RNF43 WT*, *RNF43 R117fs*, and *RNF43 G659fs*, was confirmed by inspecting the RNA-Seq reads and through Western blot validation (S3 Fig). The eVIP2 overall impact predicted the *RNF43 R117fs* variant to cause a loss of function, which is consistent with the R117fs mutation leading to a premature stop codon early in the gene, thereby disrupting the majority of the protein (Fig 2B and 2C and S3 File). Moreover, the frequent but non-specific frameshift mutations around the N-terminus near *RNF43 R117fs* also suggests it being a LOF variant (Fig 2A). Interestingly, the G659fs variant was predicted to cause a change of function with a positive impact (Fig 2B and 2C and S3 File). The G659fs frameshift occurs 126 amino acids away from the end of the wild-type gene and the termination codon in the new reading frame is 41 amino acids away from the frameshift. Frameshifts that occur late in the gene can often be assumed to be loss-of-function or not alter wild-type protein function; however, the hotspot mutational pattern of G659fs (Fig 2A) and our eVIP2 overall impact prediction (Fig 2B and 2C) suggest a change-of-function.

To evaluate if other tumor suppressor genes have hotspot frameshift mutations, we used The Cancer Genome Atlas mutation data [2]. Five tumor suppressor genes have variants *(RNF43 G659fs*, *NPM1 W288fs*, *RPL22 K15fs*, *ACVR2A K437fs*, *LARP4B T163fs)* that occur in over 5% of samples in at least one cohort (S4 Fig and S4 File). The frameshift mutations in *NPM1* and *ACVR2A* occur at the C-terminus, similar to the *RNF43 G659fs* mutation. Additionally, RNF43, ACVR2A, APC, ARID1A, GATA3, INPPL1, KMT2D, MBD6, PTEN, ZFP36L2 each had two or more frameshift mutations over 1% occurrence in a cohort (S5 Fig and S4 File). Therefore, there may be other tumor suppressor genes with multiple hotspot truncating variants that cause different functional changes and would be worth further investigation.

### Mutation-specific and WT-specific differentially expressed genes recapitulate overall LOF and COF calls

**Fig 3:**
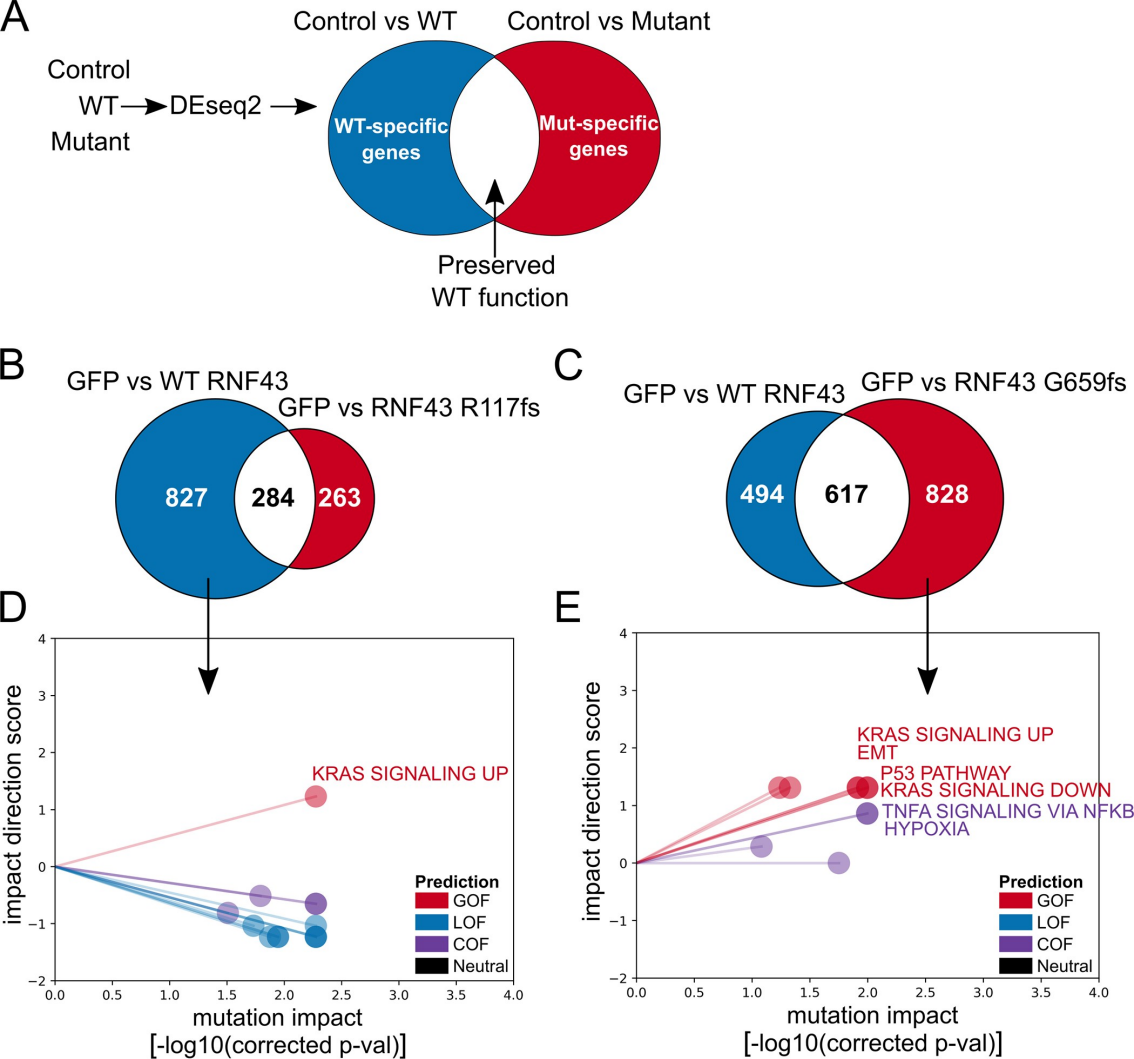
eVIP mutation-specific and WT-specific pathway analysis for LOF *RNF43 R117fs* and COF *RNF43 G659fs*. (A) Overview of method to identify WT-specific (blue) and mutation-specific (red) differentially expressed genes using DESeq2 (B) RNF43 R117fs: Count of *RNF43* WT-specific genes (blue) and *RNF43* R117fs mutation-specific genes(red) (C) RNF43 G659fs: Count of *RNF43* WT-specific genes (blue) and *RNF43 G659fs* mutation-specific genes(red) (D) Sparkler plot representation of eVIP Pathway results on *RNF43 R117fs* WT-specific genes (E) Sparkler plot representation of eVIP Pathway results on *RNF43 G659fs* mutation-specific genes.

We capitalized on the RNA-seq data and used eVIP Pathway analysis to find which pathways are impacted in each *RNF43* variant to gain further insight into the functional changes of each cancer-associated variant. To further examine the *RNF43* frameshift mutations, we defined “mutation-specific” and “WT-specific” gene sets (Fig 3A and S5–S7 Files). This allows us to find gene expression changes specific to each frameshift.

The *RNF43 R117fs* mutation had 263 mutation-specific and 827 WT-specific differentially expressed genes (Fig 3B and S5 and S6 Files). Having more WT-specific genes is consistent with *RNF43 R117fs* being a LOF mutation. The *RNF43 G659fs* mutation had 828 mutation-specific and 494 WT-specific differentially expressed genes, consistent with it being a COF mutation with a positive impact direction score (Fig 3C and S5 and S7 Files).

### eVIP Pathway analysis identifies KRAS, TNF⍺ via NFκB, and hypoxia among the top hallmark pathways impacted by *RNF43 G659fs*

While there are various tools to identify enriched pathways, eVIP Pathways is the only tool to predict pathway impact (i.e., GOF, LOF, or COF). We used the eVIP Pathways approach on the predicted *RNF43 G659fs* COF mutation and *RNF43 R117fs* mutation to investigate which specific pathways are impacted, thus giving more information on the functional role of these mutations. To find pathways impacted by each variant, eVIP Pathways method was run separately on the mutation-specific and WT-specific genes (Figs 3D and 3E and S6). We used the 50 hallmark pathway gene sets from MsigDB, to get an eVIP2 functional prediction of LOF, COF, GOF, or neutral for each tested pathway. Due to the smaller number of gene sets, we chose the 50 hallmark pathways over other databases like KEGG or Reactome for simplicity, but these other gene sets can also be used with eVIP Pathways. In order for a pathway to be characterized with eVIP Pathways, a minimum of 10 genes per pathway is required. RNF43 G659fs had 1 of the 50 hallmark pathways tested for the WT-specific genes, and *RNF43 R117fs* had 14 (S8 and S9 Files). For *RNF43 G659fs*, 10 of the 50 hallmark pathways were tested for the mutation-specific genes, however, *RNF43 R117fs* had no hallmark pathways with at least 10 mutation-specific genes (S10 File).

As expected, most impacted pathways identified from WT-specific genes for both *RNF43 R117fs* and *RNF43 G659fs* were predicted as LOF, since WT-specific genes are those that are affected by normal WT function, but not affected by the mutant (Figs 3D and S6). Unexpectedly, KRAS signaling up was predicted as a GOF pathway from WT-specific genes for *RNF43 R117fs* (Fig 3D).

The *RNF43 G659fs* mutation-specific genes had four COF pathways and six GOF pathways (Fig 3E). The four COF pathways were TNF-α signaling via NFκB, hypoxia, complement, and glycolysis. The six GOF pathways were KRAS signaling down, KRAS signaling up, P53, epithelial mesenchymal transition (EMT), IL2 Stat5 signaling, and estrogen response early. The KRAS signaling up and down pathways represent genes that are upregulated and downregulated by KRAS activation.

Little is known about these *RNF43* variants and what pathways they affect. The MSigDB “HALLMARK_WNT_BETA_CATENIN_SIGNALING” was not predicted as an impacted pathway from our analysis and is consistent with a previous report of G659fs maintaining its function as a negative regulator of Wnt [30]. In both *RNF43 R117fs* and *RNF43 G659fs* the KRAS signaling up pathway is GOF, which may suggest altered RNF43 function affects KRAS signaling.

### Reporter assays and immunoblot analysis validate eVIP2 GOF predictions for RNF43 G659fs

**Fig 4:**
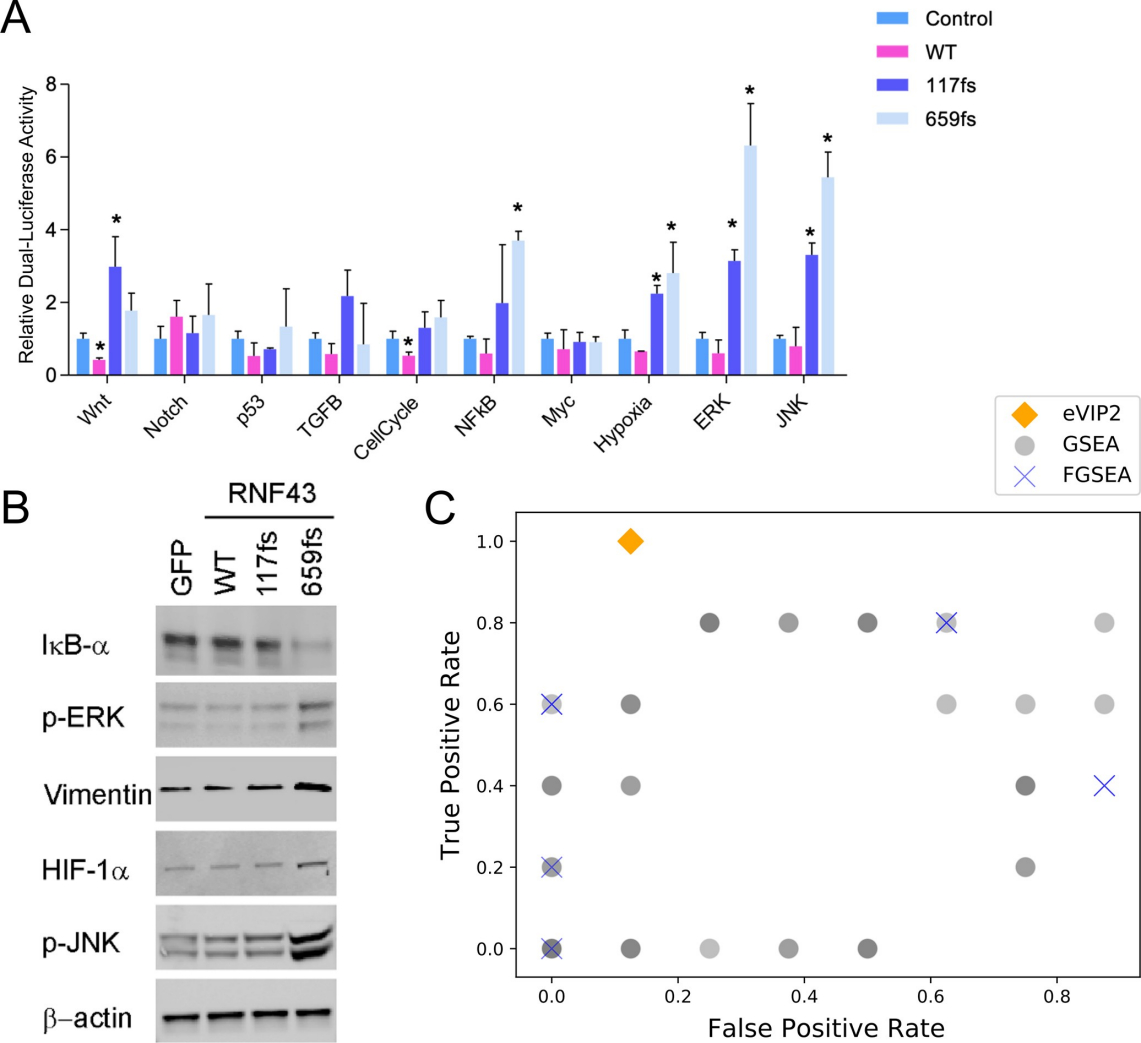
Validation of RNF43 G659fs pathway impact. (A) Cignal 10-Pathway Reporter Array data obtained in HEK-293T cells upon overexpression of Vector, RNF43-WT, *RNF43-R117fs* and *RNF43-G659fs*. Each bar represents the mean±s.d. acquired from three independent experiments. A two-tailed Student’s t-test was used for statistical analysis (*P<0.05). (B) Western blot analysis upon overexpression of GFP, RNF43-WT, RNF43-R117fs, or RNF43-G659fs in HEK-293T of IκBα, p-Erk, Vimentin, HIF-1α and p-JNK. Anti-β-actin was used as a loading control (C) True positive rate (sensitivity) and false positive rate (1-specificity) across eVIP2, 80 variations of GSEA, and 8 variations of FGSEA.

We sought to validate the impact of the *RNF43* variants, specifically *RNF43 G659fs’* impact on the six most significant mutation-specific eVIP2 pathways- KRAS signaling down, P53, TNF-α via NFκB, hypoxia, KRAS signaling up, and EMT. The Cignal 10-Pathway Reporter Array was used to measure the activity of Wnt, Notch, P53/DNA Damage, Cell Cycle/pRB-E2F, NFκB, Myc/Max, Hypoxia, MAPK/ERK, and MAPK/JNK signaling pathways (S1 Table) in HEK-293T cells co-transfected with empty vector, *RNF43 WT*, *RNF43 R117fs*, or *RNF43 G659fs* (Fig 4A and S2 Table). NFκB, hypoxia, MAPK/ERK and MAPK/JNK signaling pathways were upregulated in the presence of *RNF43 G659fs* compared with the control vector, but had no significant differences in *RNF43* WT transfected cells (Fig 4A and S2 Table). These results validate our eVIP Pathways analysis and are consistent with the finding that the *RNF43 G659fs* is a change-of-function mutation. (S1 and S2 Tables).

Interestingly, introduction of *RNF43 R117fs* also activated hypoxia, MAPK/ERK, and MAPK/JNK, although at a lesser extent than *RNF43 G659fs* (Fig 4A and S2 Table). The KRAS signaling up pathway was predicted as GOF from our eVIP Pathway analysis of *RNF43 R117fs*, which is consistent with MAPK/ERK and MAPK/JNK activation seen in the reporter assay (Figs 3D and 4A). RNF43 WT appeared effective at inhibiting Wnt, which is expected due to its known role as a suppressor of Wnt-signaling [25]. Although the Wnt pathway was not predicted to be impacted by the *RNF43 R117fs*, it caused activation of the pathway in the reporter assay.

The p53 pathway was predicted as GOF for the *RNF43 G659fs* variant by eVIP Pathway but was not significantly altered by the assay when compared to the control vector. A possible explanation for this may be an overlap between the mutation-specific genes in the p53 pathway and other pathways. Of the 14 *RNF43 G659fs*-specific differentially expressed genes in the p53 pathway, six of the genes are also in the verified activated pathways: TNF-α signaling via NFκB, KRAS signaling up, KRAS signaling down, EMT, and hypoxia. eVIP2 generates UpSet plots for exploration of gene content overlap between pathways [31] (S7 Fig).

We further investigated the functional impact of the RNF43 mutations on predicted pathways by immunoblotting. We also included an additional pathway, EMT, that was predicted to be GOF in *RNF43 G659fs*. As shown in Fig 4B, RNF43 G659fs dramatically decreased the expression level of IκBα that results in the release and nuclear translocation of active NF-κB, while RNF43 G659fs increased the p-Erk, Vimentin, HIF-1α and p-JNK suggesting the ERK, EMT, Hypoxia, JNK pathway are activated, respectively. We did not see a change in pathway activity when introducing *RNF43 WT*. This is further validation of our eVIP Pathways predictions of pathways activated by *RNF43 659fs*. Although RNF43 R117fs activated the MAPK/ERK and MAPK/JNK pathways from the reporter assay, we did not observe an effect at the protein level (Fig 4B). Perhaps the weaker transcriptional change caused by RNF43 R117fs was insufficient to result in pathway activation (Fig 4A and 4B).

Using the reporter array and immunoblot analysis we calculated sensitivity, specificity, positive predictive value (PPV), and negative predictive value (NPV) of eVIP Pathways. The sensitivity was 100% and the specificity was 87.5% (Fig 4C). The PPV and NPV were 83.3% and 100%, respectively.

### eVIP Pathways has higher or comparable sensitivity and specificity compared to standard gene set enrichment analysis

GSEA is commonly used to understand the function of a mutation and identify affected pathways [32]. GSEA ranks genes by their expression differences between two phenotypic classes and calculates enrichment scores for each gene set, which identifies pathways with cumulative changes in gene expression that are associated with a condition. This is different from eVIP Pathways, which finds pathways that are significantly different in the mutation compared to the WT by incorporating replicate consistency and signature identity to characterize pathway impact.

A total of 80 variations of GSEA were run to account for each parameter and we used four different inputs to account for various eVIP processing steps. We evaluated the sensitivity and specificity of each run, none of which outperformed eVIP2 (Fig 4C and S11 File). 17 out of the 80 variations of GSEA runs we tested identified at least one of the KRAS hallmark pathways (“KRAS Signaling Up” or “KRAS Signaling Down”) and those 17 variations had an average sensitivity of 74% and specificity of 64%.

Single sample GSEA (ssGSEA) is another pathway enrichment analysis which functions on a sample-by-sample basis. We ran a total of 12 variations for each RNF43 G659fs replicates using the same four input types and each of the three different normalization method parameters. Due to a lack of significance values associated with each pathway we could not calculate sensitivity and specificity, but none of the activated pathways from the assay or western blots (NFKB, Hypoxia, ERK, JNK, EMT) have high normalized enrichment scores (S8 Fig and S12 File).

We also evaluated the Fast Gene Set Enrichment Analysis (FGSEA) R package for preranked gene set enrichment analysis [33,34]. FGSEA is a faster implementation of enrichment analysis and more accurately estimates low GSEA p-values by using adaptive Monte Carlo sampling [34]. Two out of the eight variations of FGSEA runs we tested identified the “KRAS Signaling Up” pathway and both variations had a sensitivity of 80% and specificity of 37.5% (Fig 4C and S2 Table and S13 File).

Finally, we compared eVIP Pathways to a gene set overlap approach where gene sets with overrepresented differentially expressed genes are identified. We used the MSigDB Investigate Gene Sets tool on the *RNF43 G659fs* mutation-specific genes [32] (S1 and S2 Tables and S14 File). For the sensitivity and specificity calculations, we excluded overlaps that were less than 10. Identical to eVIP Pathways, the MSigDB Investigate Gene Sets approach predicted each of the activated pathways and the false-positive p53 pathway, giving the same sensitivity and specificity values. Though the results are similar, eVIP Pathways has the added benefit of calling pathways as LOF, COF, and GOF, which was particularly relevant for the unexpected “KRAS Signaling Up” GOF pathway in RNF43 R117fs (Figs 3D and 4A).

Overall, our validation results suggest the eVIP Pathways approach performs similarly or better than other approaches for predicting which pathways are specifically impacted by a mutation; however, additional studies on other genes and gene variants will need to be performed for a more robust evaluation.

### Primary colorectal adenocarcinoma patient samples with RNF43 truncating mutations have similar transcriptional profiles to driver BRAF missense mutations

**Fig 5:**
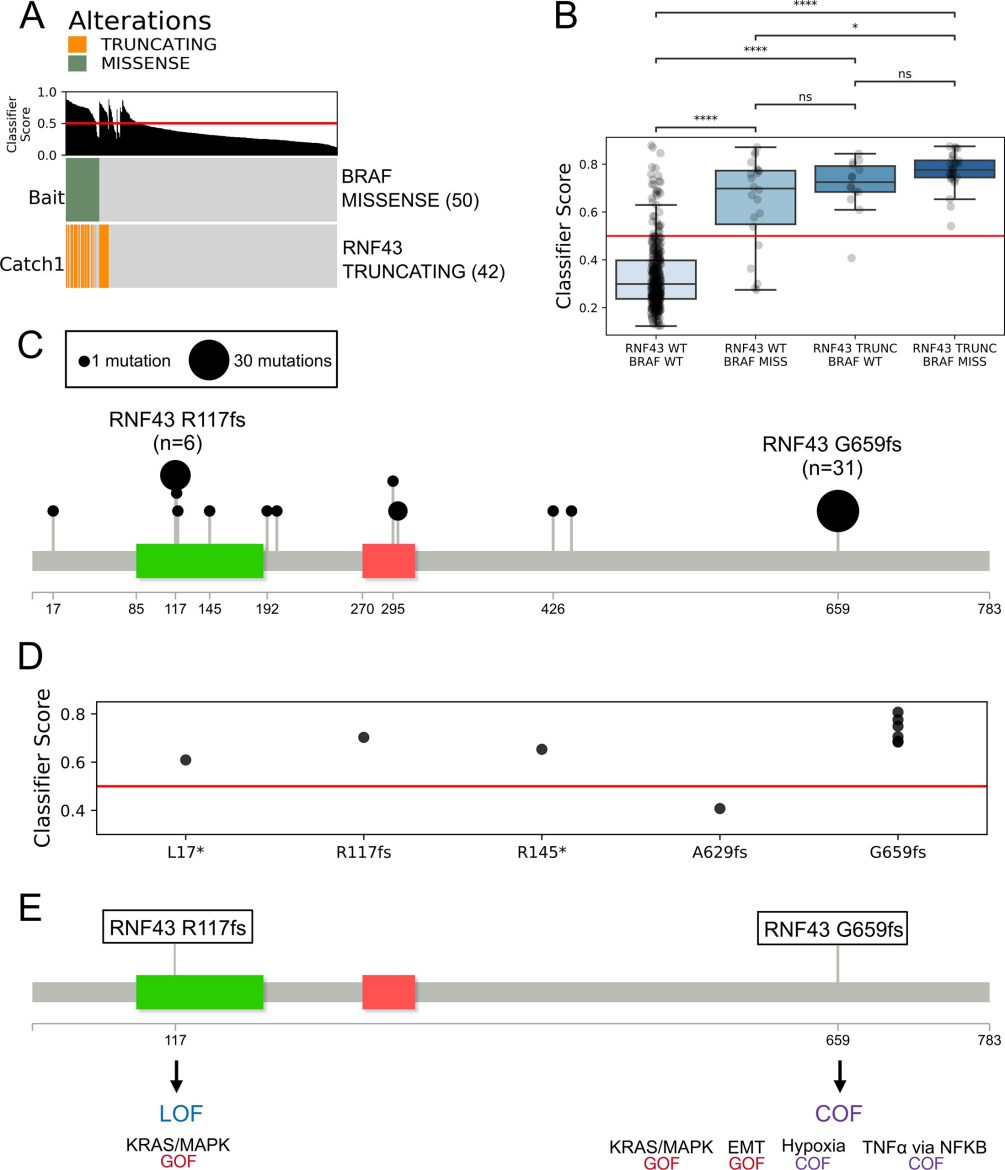
RNF43 truncating mutations have similar transcriptional profiles to BRAF missense mutations. (A) LURE oncoprint of known driver *BRAF* missense mutations, which activate the MAPK/RTK pathways, used as “bait” in TCGA COAD. LURE finds *RNF43* truncating events as a “catch” event [35]. (B) Kruskal Wallis test of LURE classifier scores across samples with different BRAF and RNF43 status. ns: 5.00e-02 < p < = 1.00e+00, *: 1.00e-02 < p < = 5.00e-02, **: 1.00e-03 < p < = 1.00e-02, ***: 1.00e-04 < p < = 1.00e-03, ****: p < = 1.00e-04) (C) Lollipop plots [20] showing the frequency RNF43-truncating mutations identified as catches (classifier score > 0.5) with LURE in the TCGA COAD cohort. The ZNRF-3 ectodomain is indicated in green and the ring finger domain is indicated in red (D) LURE classifier scores across the samples that have a single RNF43 truncating event and no co-occuring BRAF events (E) Model summarizing the predicted impacts of the *RNF43* R117fs and *RNF43 G659fs* variants.

The Learning UnRealized Events (LURE) method uses a progressive label learning framework to predict cancer driver events based on an event having a similar gene expression signature to known cancer driver events [35]. Using LURE, Haan et al. used driver BRAF missense mutations, which activate the MAPK/RTK pathway, to build a classifier and found samples with *RNF43* truncating mutations to have higher classifier scores in TCGA colorectal adenocarcinoma patients [35]. We further investigated the evidence of *RNF43* truncating mutations being associated with MAPK/RTK pathway activation in primary colorectal adenocarcinoma by incorporating the more expansive *RNF43* mutation calls identified with manual review from Giannakis et. al into the TCGA mutation status [26]. This led to the analysis of 42 samples with *RNF43* truncating events in the TCGA cohort (Fig 5A).

Though *RNF43* and *BRAF* mutations co-occur, 14 of the samples with *RNF43* truncating variants lack *BRAF* missense mutations. Samples with *RNF43* truncating variants and WT *BRAF* have strong classifier scores, suggesting that tumors with *RNF43* truncating variants activate MAPK/RTK pathways independent of *BRAF* mutations (Fig 5B). Notably, samples with both RNF43 and BRAF events have higher classifier scores than samples with only BRAF missense events.

LURE gave high Classifier Scores (>0.5) to all 6 of the samples with *RNF43 R117fs* and all 31 of the samples with *RNF43 G659fs* in the TCGA COAD cohort (Fig 5C). Some samples have multiple RNF43 truncating variants, leading the 42 samples to contain 48 RNF43 truncating events. Only one of the 48 RNF43 truncating mutations (A629fs) had a classifier score under the 0.5 cutoff and therefore is not considered a positive prediction. Ten samples had a single RNF43 truncating variant and no co-occurring BRAF mutant, among which G659fs has the highest LURE classifier scores (Fig 5D). The one resulting R117fs sample, had the next highest score, showing a potential relationship between recurrent mutations having a stronger effect.

The LURE analysis of colorectal adenocarcinoma patient samples is consistent with MAPK/ERK and MAPK/JNK being the most activated pathways in both *RNF43 R117fs* and *RNF43 G659fs* in the reporter assays (Fig 4A). The eVIP2 pathways approach also identified “KRAS Signaling Up” and “KRAS Signaling Down”, which represent genes that are upregulated and downregulated by KRAS activation, as GOF (Fig 3E). Similarly, in the *RNF43 R117fs* variant, eVIP2 identified the “KRAS Signaling Up” pathway as GOF (Fig 3D).

To further verify that the relationship LURE finds between BRAF and RNF43 variants involves the MAPK pathway, we clustered TCGA COAD samples based on their expression in key pathway genes (S9 Fig). We used the MAPK Pathway Activity Score ten gene signature [36] and found patient samples harboring RNF43 truncating mutations and/or BRAF missense mutations cluster together and have high expression in MAPK genes compared to other patient samples.

RNF43 truncating mutations co-occur with driver events in genes within the MAPK/RAS/RTK pathway, as they are common across the TCGA COAD cohort [37] (S9 Fig). Therefore, RNF43 truncating events potentially modulate MAPK/RAS/RTK signaling with other co-occurring driver mutations. We also investigated expression of the experimental marker genes from the other eVIP2 experimentally validated pathways. Within many of the RNF43 truncating mutated samples, marker genes in hypoxia (HIF1A), NFKB (NFKB1, NFKB2, REL, RELA,RELB), and EMT (VIM) also have higher expression (S9 Fig). We also included colorectal cancer EMT markers, TWIST1 and SNAI1 [38].

## Discussion

Previous work showed that high-throughput expression-based phenotyping can accurately distinguish between neutral and impactful mutations [7]. eVIP was used to functionally profile a diverse set of 194 lung adenocarcinoma alleles from 53 genes in a single assay, addressing the challenge of interpreting the millions of mutations that have been identified in cancer. In this study, we present the eVIP2 software, with the following meaningful advances and innovations:

1. Making eVIP into an easy-to-use python tool, instead of a collection of scripts
2. Allowing import of RNA-seq, instead of only L1000-specific inputs and measurements
3. eVIP Pathway-level impact analysis
4. Improved evaluation of statistical FDR thresholds

Here, we characterize overall and pathway-specific impact of two common frameshift variants in colorectal, gastric and endometrial cancers (Fig 5E). Both of the tested frameshift mutations in *RNF43* have been assumed to be loss of function [39]. We show that the two frameshift mutations actually have different effects on RNF43 gene function. While *RNF43 R117fs* was LOF, eVIP2 predicted the *RNF43 G659fs* variant to be a COF mutant.

We validated *RNF43 G659fs* COF status, showing the mutant affects the NFκB via TNF-α, hypoxia, MAPK/ERK, MAPK/JNK, and EMT pathways, which are not affected by the overexpression of WT RNF43. The five pathways differentially affected by *RNF43 G659fs* in the functional experiments were identified as COF or GOF with eVIP Pathways.

eVIP2 predicted both R117fs and G659fs variants to have a GOF in KRAS pathways. In the experimental assay, MAPK/ERK and MAPK/JNK were activated by overexpression of both variants, but not by the overexpression of WT RNF43. The effect on the pathways is stronger in G659fs than R117fs, and only in G659fs were ERK and JNK activation validated by western blots.

The MAPK pathway is commonly activated in cancers by driver mutations in KRAS, NRAS, and BRAF. LURE analysis found *RNF43* truncating mutations to have similar transcriptional signatures to BRAF mutations in colorectal adenocarcinomas [35]. Among the RNF43 truncating variants, G659fs had the highest classifier scores, followed by R117fs, which is consistent with more ERK and JNK activation in G659fs than R117fs in our expression assay. Interestingly, samples that have both RNF43 truncating variants and BRAF missense variants have higher LURE classifier scores than samples with only BRAF missense variants.

Despite activating the same pathway, *BRAF* mutant tumors have distinct expression signatures from *KRAS* mutant tumors in colon cancer [40,41]. To explore MAPK pathway expression in colon adenocarcinoma patient samples, we used the transcriptional MAPK Pathway activity score, which is a ten-gene signature that consists of MAPK target genes that measures MAPK activity across multiple tissue types independently of RAS or BRAF mutational status. Clustering of colorectal patient expression profiles showed samples with RNF43 truncating variants and BRAF missense variants cluster together and appear distinct from KRAS G12 mutated samples.

Known interplay among the validated eVIP2-identified pathways suggests a concerted oncogenic impact of *RNF43 G659fs*. In colon cancer cells, MAPK/RAS activation induces epithelial–mesenchymal transition (EMT)[42–45]. Similarly, TNF-α via NF-κΒ has been found to induce EMT in colorectal cancer cells and other carcinomas[46–49] and additionally have extensive cross talk with hypoxia [50]. In many RNF43 frameshift mutant TCGA colon adenocarcinoma patient samples, we found activation of EMT, NFKB, and hypoxia marker genes.

In contrast to eVIP2’s high sensitivity, none of the 80 GSEA runs reached the same level of sensitivity. When using the mutation-specific genes generated from eVIP2, the GSEA Investigate Gene Sets overlap tool identified the same pathways as eVIP2. However, eVIP2 has the benefit of providing information about the directionality of the change (gain or loss of function), which is particularly relevant in the KRAS signaling GOF call in the LOF R117fs variant (Fig 3D).

A strength of the eVIP2 approach is that it can be applied to any mutation and does not require prior knowledge of the gene. A recent study claims *RNF43 G659fs* is a passenger mutation, which is not supported by our results or the Haan et al. results [30,35]. Tu et al. based their conclusion that RNF43 G659fs is unlikely to play a role in tumorigenesis based on its effects on the Wnt pathway [30]. This is consistent with this study, where we did not find the Wnt pathway to be impacted by *RNF43 G659fs* by eVIP Pathway analysis. However, Tu et. al. mainly focused on the effect on the mutation’s involvement in the Wnt pathway. With eVIP Pathways, we can profile multiple pathways at once, which is especially helpful when investigating mutations in genes that are not well characterized. We found that *RNF43 G659fs* has a functional impact on other pathways and is unlikely to be a passenger mutation.

It is important for future work to investigate the impact of RNF43 variants in additional cellular context as we have previously shown that this can change the predicted impact [7]. These additional analyses would be straightforward with our eVIP2 computational workflow.

The eVIP2 software uses gene expression data from L1000 expression profiling or RNA-seq to predict overall mutation and pathway impact. eVIP Pathways is flexible and can be used with custom gene sets or from existing gene sets from MsigDB, KEGG, or Reactome. Since the original description of the eVIP algorithm, we have improved the software to be more easily run by others to perform similar analyses, thus making this approach more available for mutation profiling by others in the scientific community.

### Availability and future directions

eVIP2 is implemented in Python. The software, instruction manual, and example data are available on GitHub (https://github.com/BrooksLabUCSC/eVIP2). RNA-Seq data are recommended as input; however, any molecular profiling data (e.g., L1000, pre-processed gene expression) can be used as input. For future versions of the software, we will test eVIP2 on other molecular profiling such as alternative splicing signatures to investigate the effects of cancer-associated variants.

## Methods

### A549 empty vector samples and ARAF variant functional impact prediction

Cell lysates were generated in quadruplicate and as previously described for A549 lines transfected with WT *ARAF*, *ARAF V145L*, *ARAF S214F*, *ARAF S214F/D429A*, and *ARAF S214C/D429A*, in addition to an empty control (Berger et al. 2016). Cell lysates were stored in TCL buffer (Qiagen). RNA was purified using SMART-Seq v4 Ultra Low Input RNA Kit for Sequencing (Takara Bio), optimized in-house for diverse transcript sets. Libraries were constructed using the Illumina Nextera XT for 75bp paired-end reads and then pooled and sequenced on the Illumina HiSeq platform (Fred Hutch Genomics Core). Transcripts were quantified with Kallisto (v0.46.2) with index built on the GRCh38 transcriptome. eVIP2 was then run using default parameters.

### RNF43 variant functional impact prediction

Quadruplicate transfections of GFP, WT *RNF43*, *RNF43 R117fs*, and *RNF43 G659fs* were done in HEK-293T cells and sequenced with NextSeq 500 (75 nucleotide reads, single end). The Qiagen RNEasy protocol, including DNAse I treatment and NEBNext library construction protocol for Illumina were used. S3 Fig showing validation of WT and variant expression was made using Integrative Genome Viewer [51]. Transcript counts were generated using Kallisto [10]. The Kallisto index was built from Ensembl release 94 GRCh38 cDNA transcriptome. Kallisto counts were imported to DESeq2 using tximport and DESeq2 was run using default parameters [15]. eVIP2 was run using default parameters:

-use_c_pval = True

-min_genes = 10

-min_tpm = 1

-conn_thresh = 0.1

-mut_wt_rep_thresh = 0.1

-disting_thresh = 0.1

-mut_wt_rep_rank_diff = 0

-cond_max_diff_thresh = 0.2

### Tumor suppressor gene mutation frequency analysis

We used cbioportal to access the mutation data for each of the tumor suppressor genes defined by Davoli et al. [52–54]. Frameshift mutation frequency was calculated within each TCGA pan cancer cohort and DFCI cohort, totaling 13216 samples from 41 studies[4,22,55–57]. We Identified 5 tumor suppressor genes with recurrent mutations with a frequency over 5% within a cohort and 10 tumor suppressor genes with at least 2 frameshift variants over 1%. We excluded mutations with a count under 5. Lollipop diagrams were constructed using the Lollipop tool[20](S4 and S5 Figs). For each gene, we only visualize the cohorts in which a mutation had a high frequency (1% or 5%).

### L1000 correlation methods comparison

For the comparison of L1000 weighted connectivity score versus Spearman correlation, eVIP was run using default parameters. The scatter plot in S1 Fig uses the corrected p-value (“wt_mut_rep_vs_wt_mut_conn_c_pval”) from the Kruskal-Wallis Test.

### Cignal finder cancer 10-pathway reporter array

The Cignal pathway reporter assay (SABiosciences/Qiagen, Frederick, MD, USA, Cat. No. CCA-001L/336821) was performed following the instructions provided by the manufacturer. Briefly, 4000 cells/well of HEK-293T cells were seeded in 96-well plates and allowed to settle overnight in a 37°C incubator with 5% CO_2_ before transfection. 100 ng of each Cignal dual-luciferase reporter constructs with 200 ng of *RNF43* variants or empty vector constructs were co-transfected into the cells by using Lipofectamine LTX (Thermo Fisher Scientific, Inc.). After 48 hours transfection, cells were harvested and measured the dual-luciferase activities based on Firefly-to-Renilla luminescence ratio using the Dual-Luciferase Reporter Assay System (Promega, Madison, WI, USA).

### Western blot analysis

HEK293T cells infected with *RNF43* WT, R117fs or G659fs fusion with V5-tag were harvested in RIPA Buffer (Sigma Aldrich, Cat. No. #R0278) supplemented by Protease Inhibitor Cocktail (Cell Signaling, Cat. No. #5871), then resolved by 10% SDS-PAGE. Western blot analysis was performed by the standard method. The protein expression of *RNF43* WT and mutants was detected by the primary antibodies anti-V5 Tag (1:5,000, mouse, Monoclonal, Life Technologies, Cat. No. R96025) and anti-β-actin (1,2,000, rabbit, polyclonal, Cell Signaling, Cat. No. #4970) was used as a loading control. Goat anti-Mouse and goat anti-rabbit secondary antibody were obtained from Licor and used at 1:15000 dilution. The proteins of interest were visualized using a two-color Li-COR Odyssey Imager (LI-COR).

### Gene Set Enrichment Analysis (GSEA) and Single Sample Gene Set Enrichment Analysis (ssGSEA)

Gene set enrichment analysis was performed in python with GSEAPy [12,32]. For consistency with eVIP Pathways, we used the same 50 Hallmark pathways (h.all.v6.0.symbols.gmt) and required at least 10 genes for each gene set. We performed two GSEA comparisons: (1) GFP vs *RNF43* G659fs (2) *RNF43* WT vs *RNF43* G659fs. To account for different eVIP2’s processing steps, we used four types of input data: (1) gene TPM counts, (2) filtered and log2 transformed gene TPM counts, (3) filtered, log2 and z-transformed gene TPM counts, (4) mutation-specific genes from filtered and log2 transformed gene TPM counts. We evaluated both permutation types (1)“gene_set” and (2) “phenotype” and all five GSEA methods for used to rank samples (1) “signal_to_noise”, (2) “t_test”, (3) “ratio_of_classes”, (4) “diff_of_classes”, (5) “log2_ratio_of_classes”. Pathways with a FDR under 0.25 were considered significant. The results for the 80 GSEA runs are available in S11 File.

We used the GSEAPy ssGSEA function to run ssGSEA, with the same four inputs and each of the three sample normalization method parameters (1) "rank”, (2) "log", (3) "log_rank". The resulting normalized enrichment scores of the 48 ssGSEA runs (12 for each of the 4 *RNF43* G659fs replicates) are available in S12 File.

### Fast Gene Set Enrichment Analysis (FGSEA)

Fast Gene Set Enrichment Analysis (FGSEA) is an R tool that performs preranked gene set enrichment analysis. FGSEA requires a pre-ranked file gene file, that we created from DESeq2’s Wald statistic in two comparisons: (1)GFP vs *RNF43* G659fs and (2)*RNF43* WT vs *RNF43* G659fs. We evaluated the (1)“fgsea” and (2)“fgseaMultilevel” functions and ranked gene input of (1) all genes and (2) mutation-specific genes. Pathways with an adjusted p-value under 0.05 were considered significant. The results for the 8 runs of FGSEA are available in S13 File.

### MSigDB investigate gene sets tool

The list of *RNF43* G569fs mutation-specific genes were input to the MSigDB Investigate Gene Sets tool to compute overlaps in the Hallmark gene set (http://www.gsea-msigdb.org/gsea/msigdb/annotate.jsp) [12,32]. The results are in S14 File.

### TCGA Learning UnRealized Events (LURE) analysis

We used the Learning UnRealized Events (LURE) method using BRAF missense as the bait event within the TCGA COAD cohort [35]. Default parameters were used. RNF43 mutation statuses from Giannakis et al [26] were incorporated into the TCGA mutation calls.

### TCGA COAD patient clustering

TCGA COAD RNA-seq gene-level transcription estimates (log2(x+1) transformed RSEM normalized counts) and clinical data (MSI status) were obtained from the cBioPortal [21,54]. MAPK/RAS/RTK event status was determined using genes defined by Sanchez-Vega et al. [37]. We used cbioportal to determine samples harboring events classified as drivers (Mutations, Fusions, Copy Number Alterations) in any of the 85 genes. Hierarchical clustering was done using the Ward method.

## Supporting information

S1 Fig Comparing eVIP correlation metrics. Comparison of eVIP p-values when using Spearman rank correlation values or weighted connectivity scores (wtcs) as input. The dotted horizontal and vertical line represents p-value cutoff of .05.
(TIFF)

S2 Fig Sparkler plot representation of functional impact of ARAF variants. Sparkler plot representation of (A) overall eVIP2 results on ARAF variants and (B) ARAF variants using only genes from the L1000 assay.
(TIFF)

S3 Fig Validation of expression of RNF43 frameshift variants. (A) HEK293T cells transfected with RNF43 WT and mutants were verified by western blotting. V5 antibody (Red) indicated RNF43 overexpression, β-Actin (Green) used as control. (B) Expression of RNF43 WT, RNF43 R117fs, and RNF43 G659fs from RNA-.
(TIFF)

S4 Fig Tumor suppressor genes with a frameshift mutation at frequency of 5% or higher. Lollipop diagrams showing the frequency of mutations in tumor suppressor genes with a frequency of at least 5% [20]. For each gene, only mutation counts from cohorts the variants have a frequency of at least 1% in are shown.
(TIFF)

S5 Fig Tumor suppressor genes with two frameshift mutations with frequency of 1% or higher. Lollipop diagrams showing the frequency of mutations in tumor suppressor genes with at least two frameshift mutations with a frequency of 1% within a TCGA or DFCI cohort [20]. For each gene, only mutation counts from cohorts the variants have a frequency of at least 1% in are shown.
(TIFF)

S6 Fig *RNF43 G659fs* WT-specific eVIP Pathway. Sparkler plot representation of eVIP Pathways results using *RNF43 G659fs* WT-specific genes.
(TIFF)

S7 Fig Gene content overlap in *RNF43 G659fs* mutation-specific pathways. Upset plot generated using eVIP2 and the UpSetPlot Python package [31].
(TIFF)

S8 Fig ssGSEA normalized enrichment scores in the 13 validated Hallmark pathways. Distribution of normalized enrichment scores for each of the 12 variations of ssGSEA runs for each of the 4 RNF43 G659fs replicate across the validated Hallmark pathways.
(TIFF)

S9 Fig TCGA COAD expression in marker genes from eVIP2-identified pathways. (A) Hierarchical clustering of gene expression (z-score) across TCGA COAD samples using pathway marker genes. The top color bar shows the LURE BRAF missense mutation score, BRAF missense status, RNF43 truncating status, RNF43 G659fs status, driver event status for genes in the RTK-RAS-MAPK pathway [37], KRAS G12(A,C,D,R,S or,V) status, and microsatellite instability status (high, low, or stable) status. The side color bar shows what pathway each gene is a marker for. (B,C) ETV5 and DUSP4 expression (log2(x+1) transformed RSEM normalized counts) across RNF43 and BRAF status. (Kruskal Wallis test across groups. ns: 5.00e-02 < p < = 1.00e+00, *: 1.00e-02 < p < = 5.00e-02, **: 1.00e-03 < p < = 1.00e-02, ***: 1.00e-04 < p < = 1.00e-03, ****: p < = 1.00e-04) (D) Subset of clustering from (A) showing only samples with RNF43 G659fs (and no other truncating RNF43 variants) with WT BRAF.
(TIFF)

S1 Table Cignal Reporter Assay used to measure pathway activity and their associated hallmark pathways. (DOCX)

S2 Table Reporter assay results compared with eVIP Pathways and GSEA. Pathway reporter assay results are more consistent with eVIP Pathway prediction than with GSEA. Results from a pathway reporter array using different sample group comparisons (first 3 columns). Pathways were considered significant with p-value under .05, then the direction of difference is reported as inhibited or activated. The last two columns are eVIP Pathway and GSEA Investigate Gene Sets results (GOF = Gain of Function, COF = Change of function). GSEA Investigate Gene Sets was run on RNF43 G659fs mutation-specific genes. Only significant pathways (FDR q-value < .05) that contain at least 10 genes in the overlap are shown.
(DOCX)

S1 File Overall eVIP2 outputs for *ARAF* variants. Text file containing eVIP2 overall functional predictions for *ARAF* variants.
(TXT)

S2 File Overall eVIP2 outputs for *ARAF* variants using only L1000 genes. Text file containing eVIP2 overall functional predictions for *ARAF* variants when eVIP2 is run only using genes from the L1000 assay.
(TXT)

S3 File Overall eVIP2 outputs for *RNF43* variants. Text file containing eVIP2 overall functional predictions for *RNF43 R117fs* and *RNF43 G659fs*.
(TXT)

S4 File Frequency of frameshift mutations in tumor suppressor genes within TCGA and DFCI cohorts. Text file containing each frameshift mutation in a tumor suppressor gene and its count and frequency within a patient cohort.
(CSV)

S5 File DESeq2 GFP vs *RNF43 WT*. Text file containing DESeq2 outputs for GFP replicates vs *RNF43 WT* replicates.
(CSV)

S6 File DESeq2 GFP vs *RNF43 R117fs*. Text file containing DESeq2 outputs for GFP replicates vs *RNF43 R117fs* replicates.
(CSV)

S7 File DESeq2 GFP vs *RNF43 G659fs*. Text file containing DESeq2 outputs for GFP replicates vs *RNF43 G659fs* replicates.
(CSV)

S8 File eVIP Pathways WT-specific genes for *RNF43 R117fs*. Text file containing eVIP Pathways results for *RNF43 G659fs* using WT-specific differentially expressed genes.
(TXT)

S9 File eVIP Pathways WT-specific genes for *RNF43 G659fs*. Text file containing eVIP Pathways results for *RNF43 G659fs* using WT-specific differentially expressed genes.
(TXT)

S10 File eVIP Pathways mutation-specific genes for *RNF43 G659fs*. Text file containing eVIP Pathways results for *RNF43 G659fs* using mutation-specific differentially expressed genes.
(TXT)

S11 File GSEA results. Text file containing GSEA results from various runs.
(CSV)

S12 File ssGSEA results. Text file containing ssGSEA results from various runs.
(CSV)

S13 File FGSEA results. Text file containing FGSEA results from various runs.
(CSV)

S14 File GSEA Investigate Gene Sets with *RNF43 G659fs* mutation-specific genes. Text file containing results from GSEA Investigate Gene Sets tool using the *RNF43 G659fs* mutation-specific genes.
(TSV)
