# Supplementary figures and images for: eVIP2: Expression-based variant impact phenotyping to predict the function of gene variants
Source: PLoS Comput Biol. 2021 Jul 2;17(7):e1009132. doi: 10.1371/journal.pcbi.1009132 (PMC8281988; doi:10.1371/journal.pcbi.1009132)

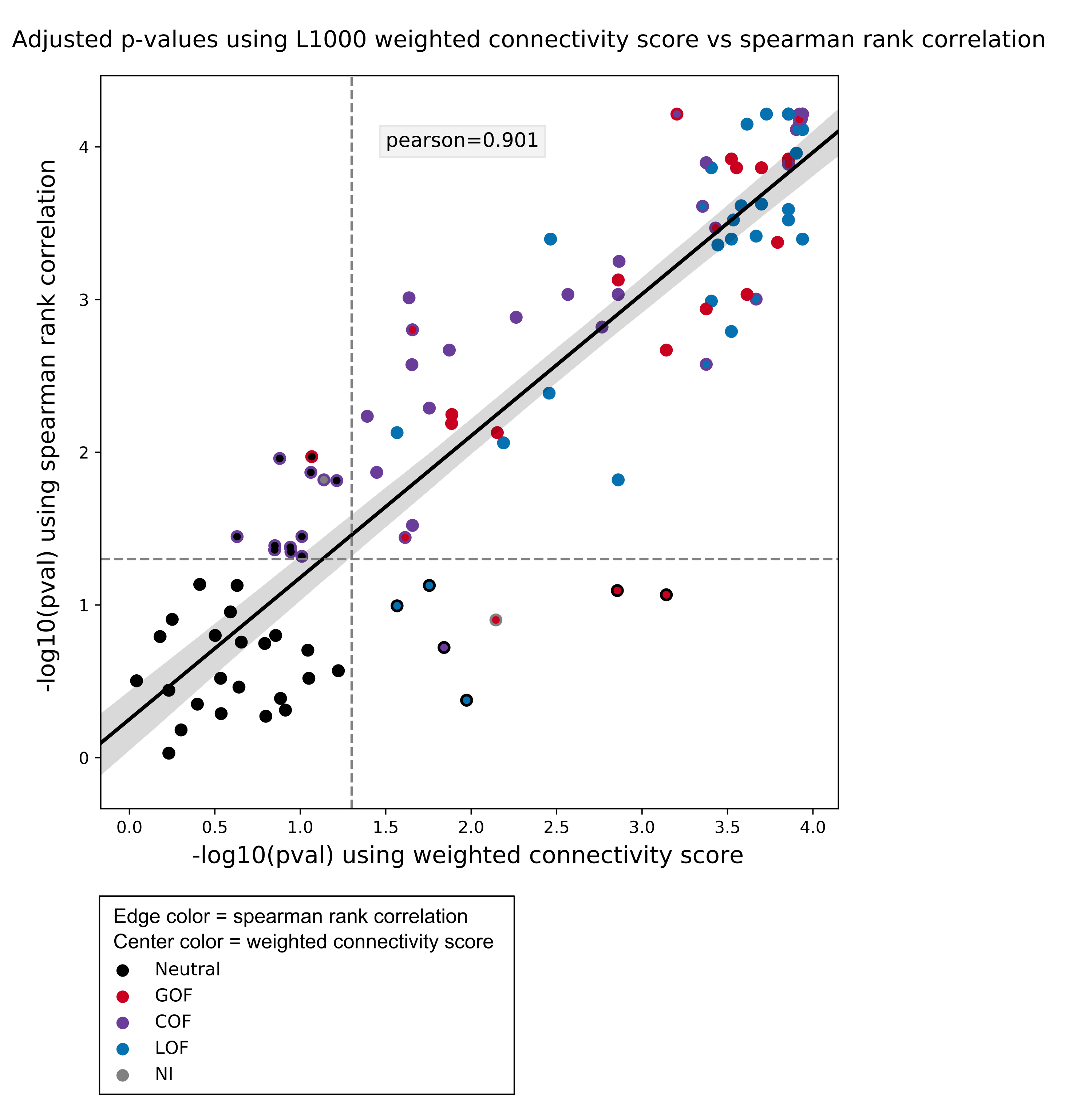

Supplement: S1 Fig — Comparison of eVIP p-values when using Spearman rank correlation values or weighted connectivity scores (wtcs) as input. The dotted horizontal and vertical line represents p-value cutoff of .05. (TIFF) [file pcbi.1009132.s001.tiff]

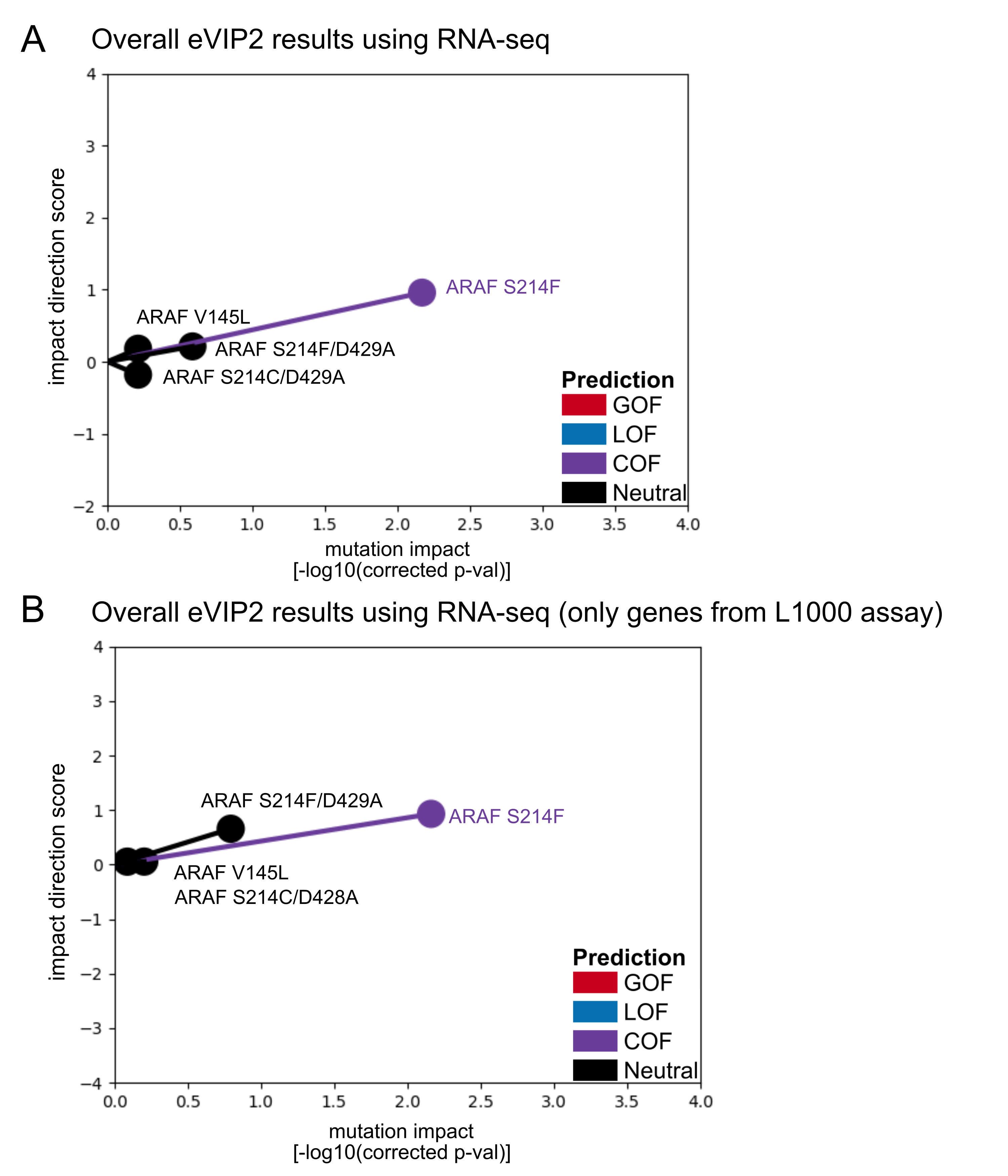

Supplement: S2 Fig — Sparkler plot representation of (A) overall eVIP2 results on ARAF variants and (B) ARAF variants using only genes from the L1000 assay. (TIFF) [file pcbi.1009132.s002.tiff]

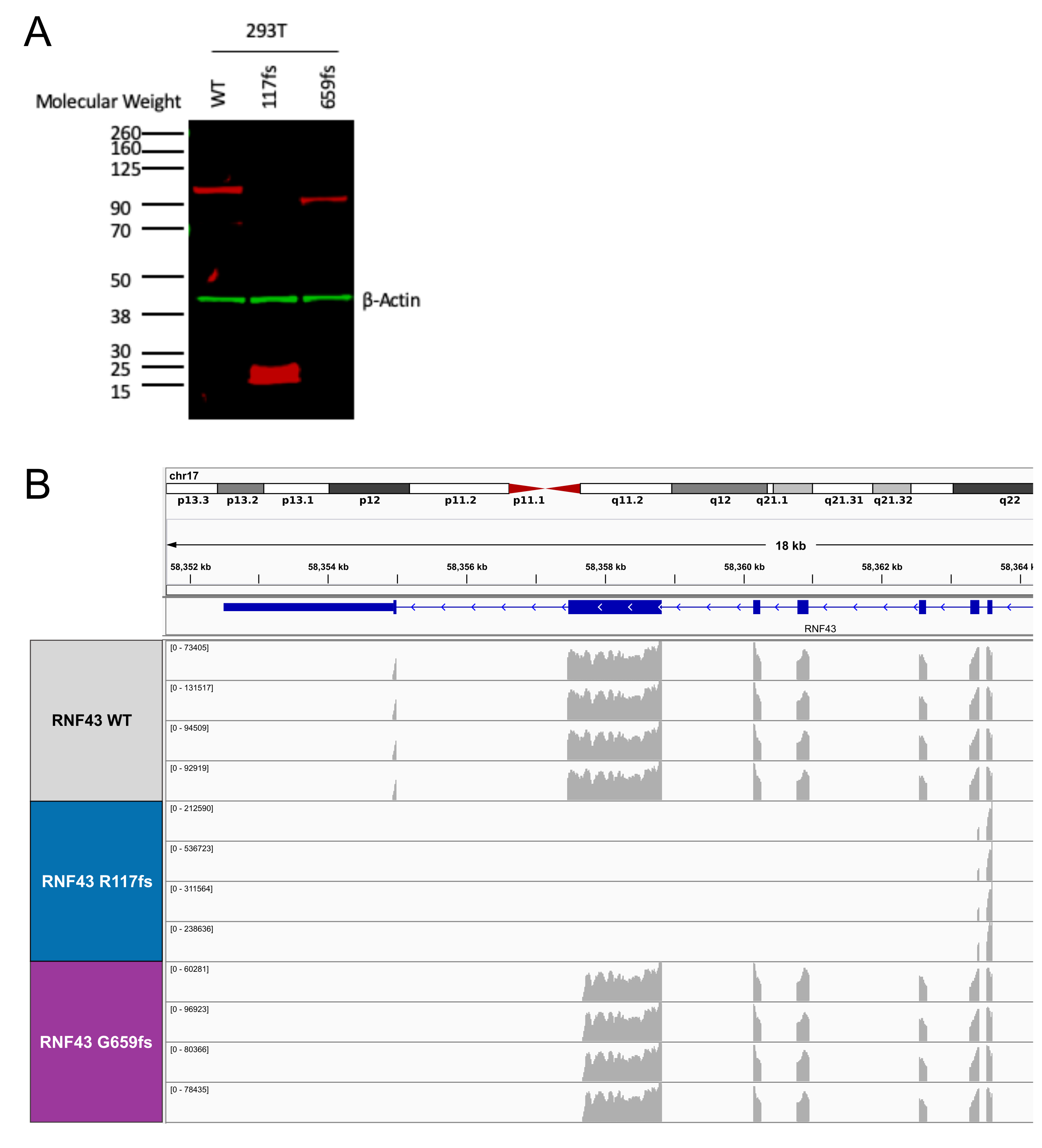

Supplement: S3 Fig — (A) HEK293T cells transfected with RNF43 WT and mutants were verified by western blotting. V5 antibody (Red) indicated RNF43 overexpression, β-Actin (Green) used as control. (B) Expression of RNF43 WT, RNF43 R117fs, and RNF43 G659fs from RNA-. (TIFF) [file pcbi.1009132.s003.tiff]

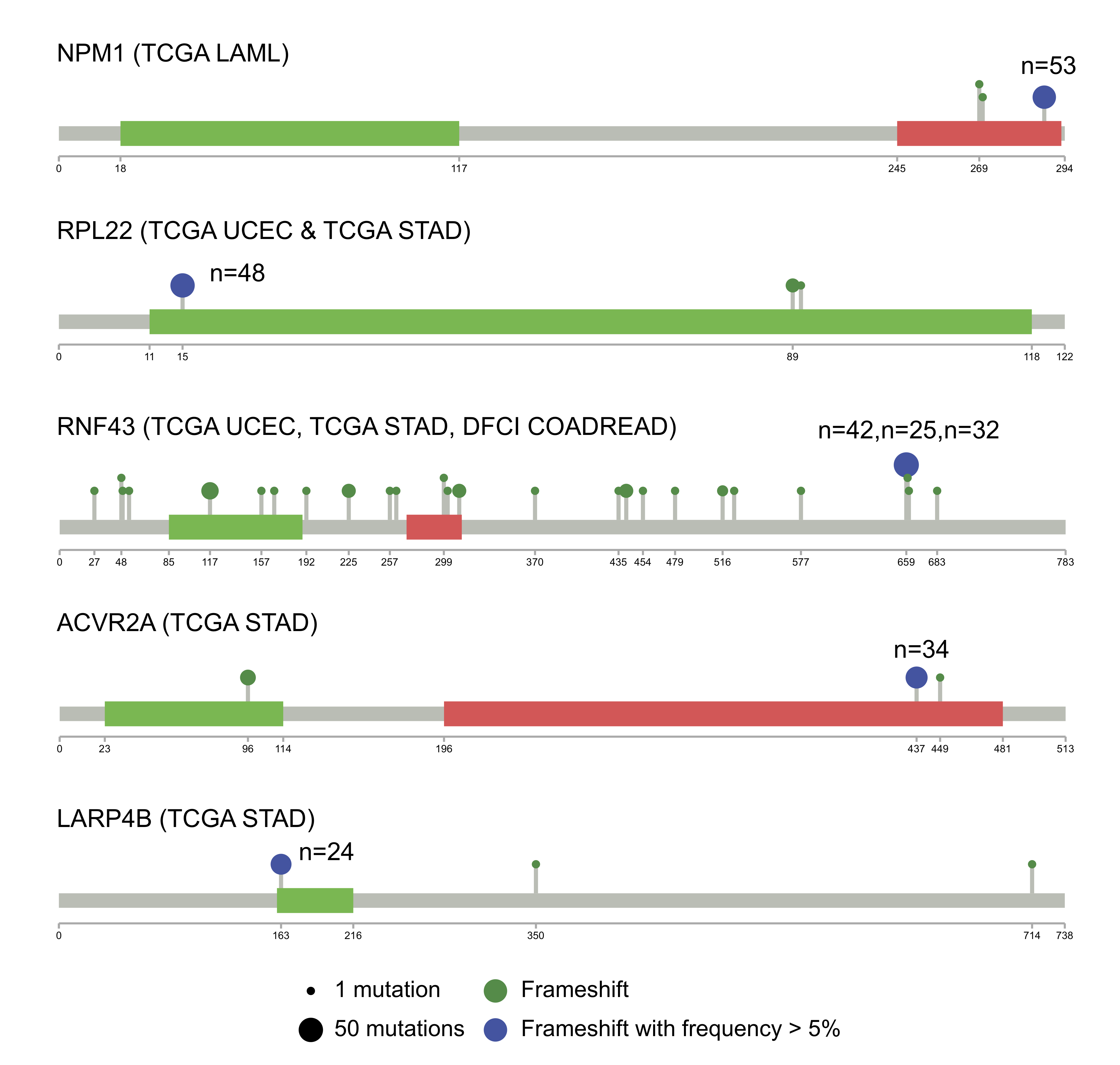

Supplement: S4 Fig — Lollipop diagrams showing the frequency of mutations in tumor suppressor genes with a frequency of at least 5% [20]. For each gene, only mutation counts from cohorts the variants have a frequency of at least 1% in are shown. (TIFF) [file pcbi.1009132.s004.tiff]

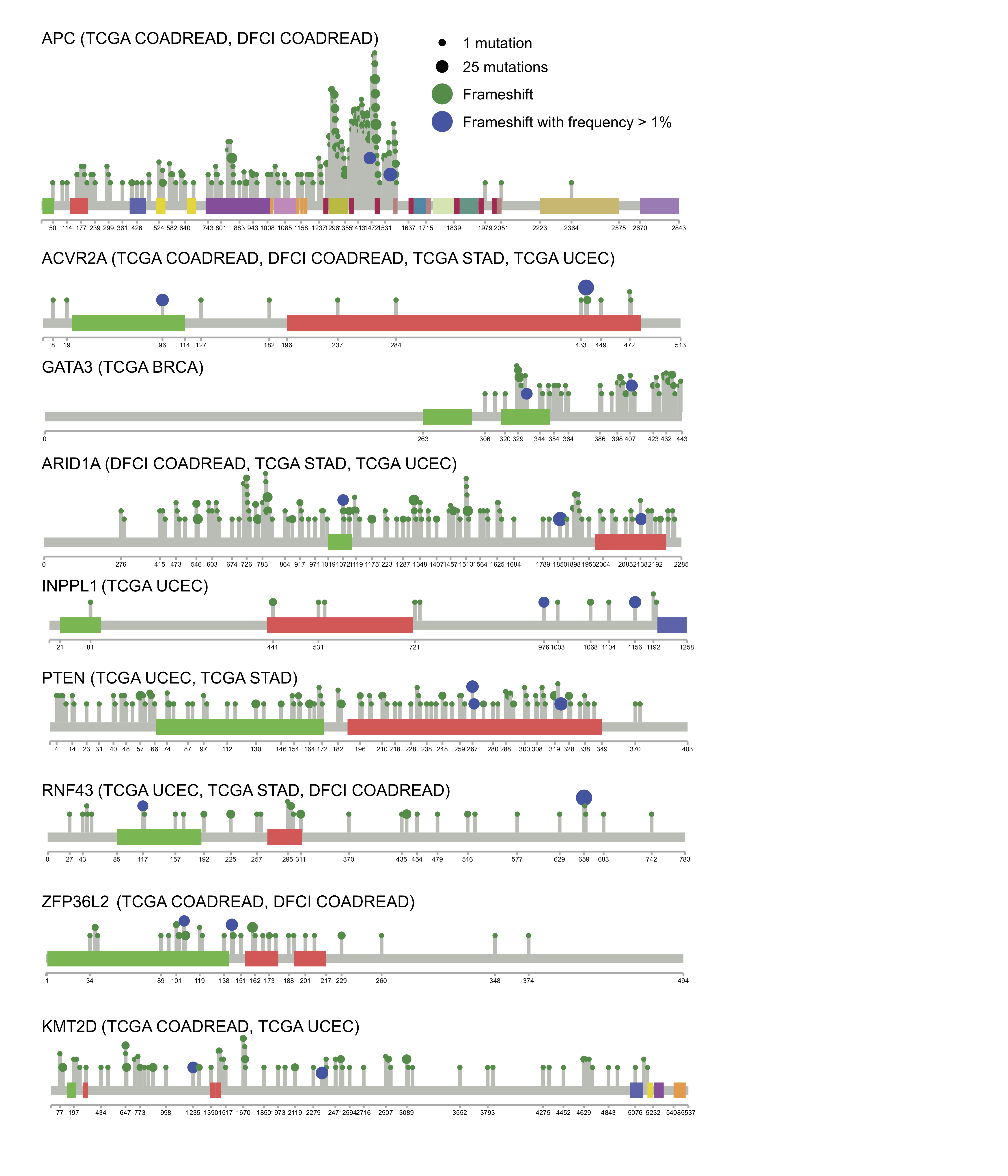

Supplement: S5 Fig — Lollipop diagrams showing the frequency of mutations in tumor suppressor genes with at least two frameshift mutations with a frequency of 1% within a TCGA or DFCI cohort [20]. For each gene, only mutation counts from cohorts the variants have a frequency of at least 1% in are shown. (TIFF) [file pcbi.1009132.s005.tiff]

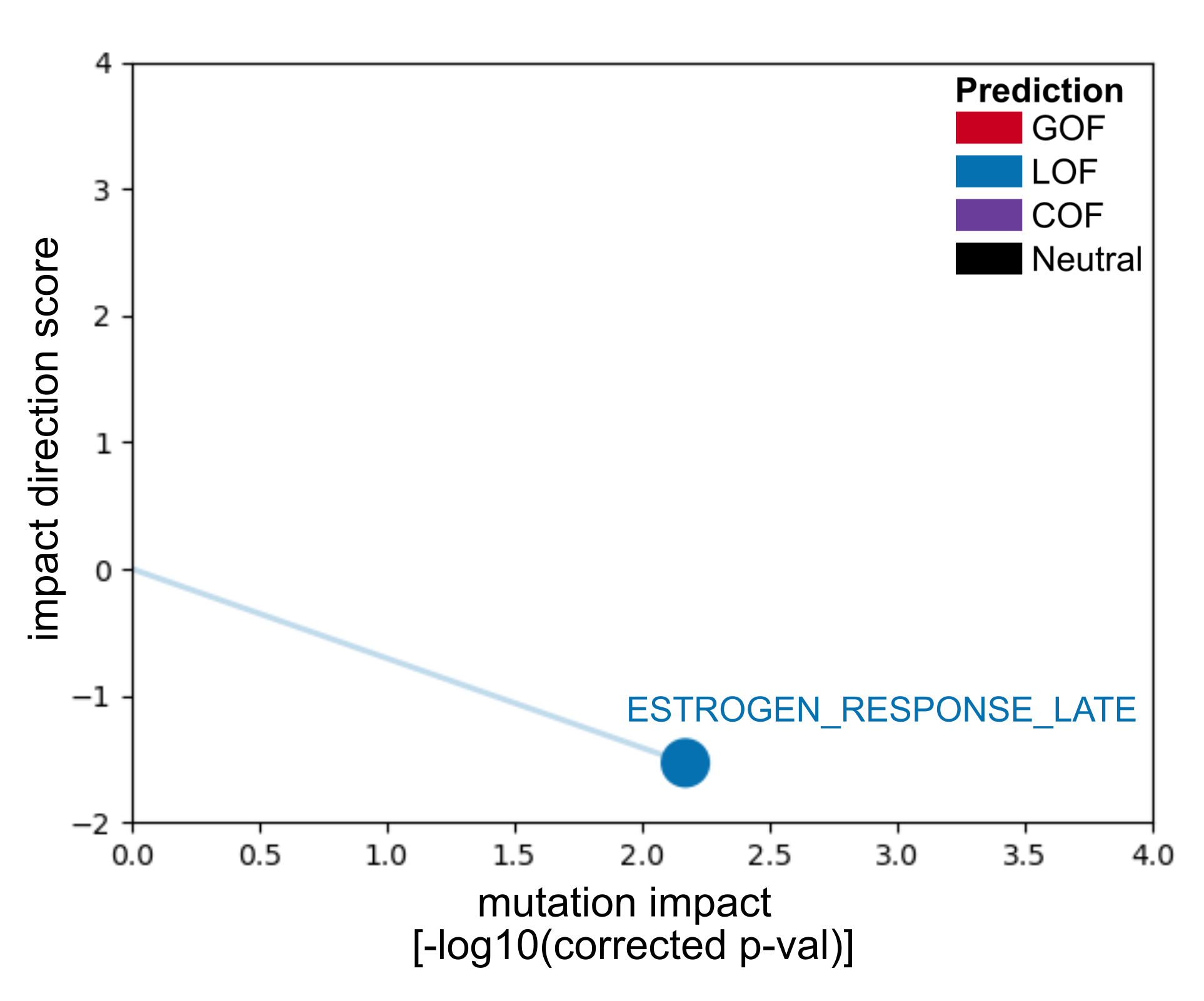

Supplement: S6 Fig — Sparkler plot representation of eVIP Pathways results using RNF43 G659fs WT-specific genes. (TIFF) [file pcbi.1009132.s006.tiff]

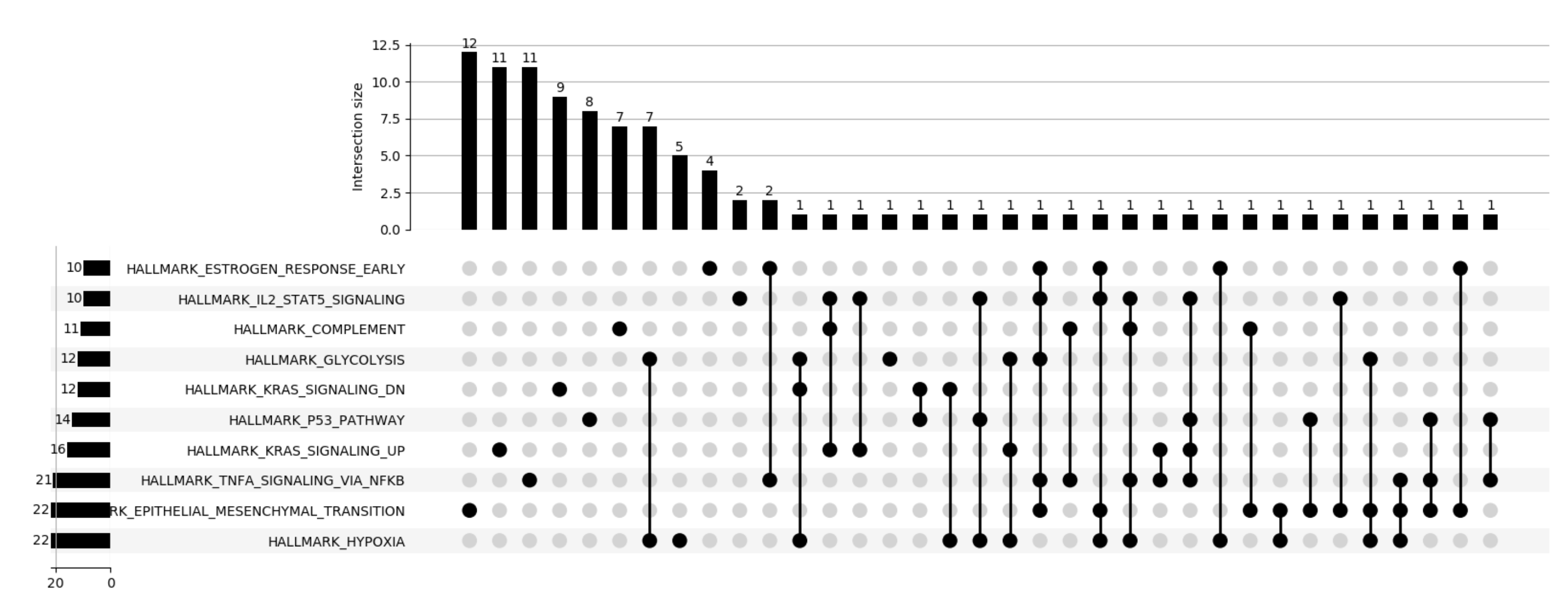

Supplement: S7 Fig — Upset plot generated using eVIP2 and the UpSetPlot Python package [31]. (TIFF) [file pcbi.1009132.s007.tiff]

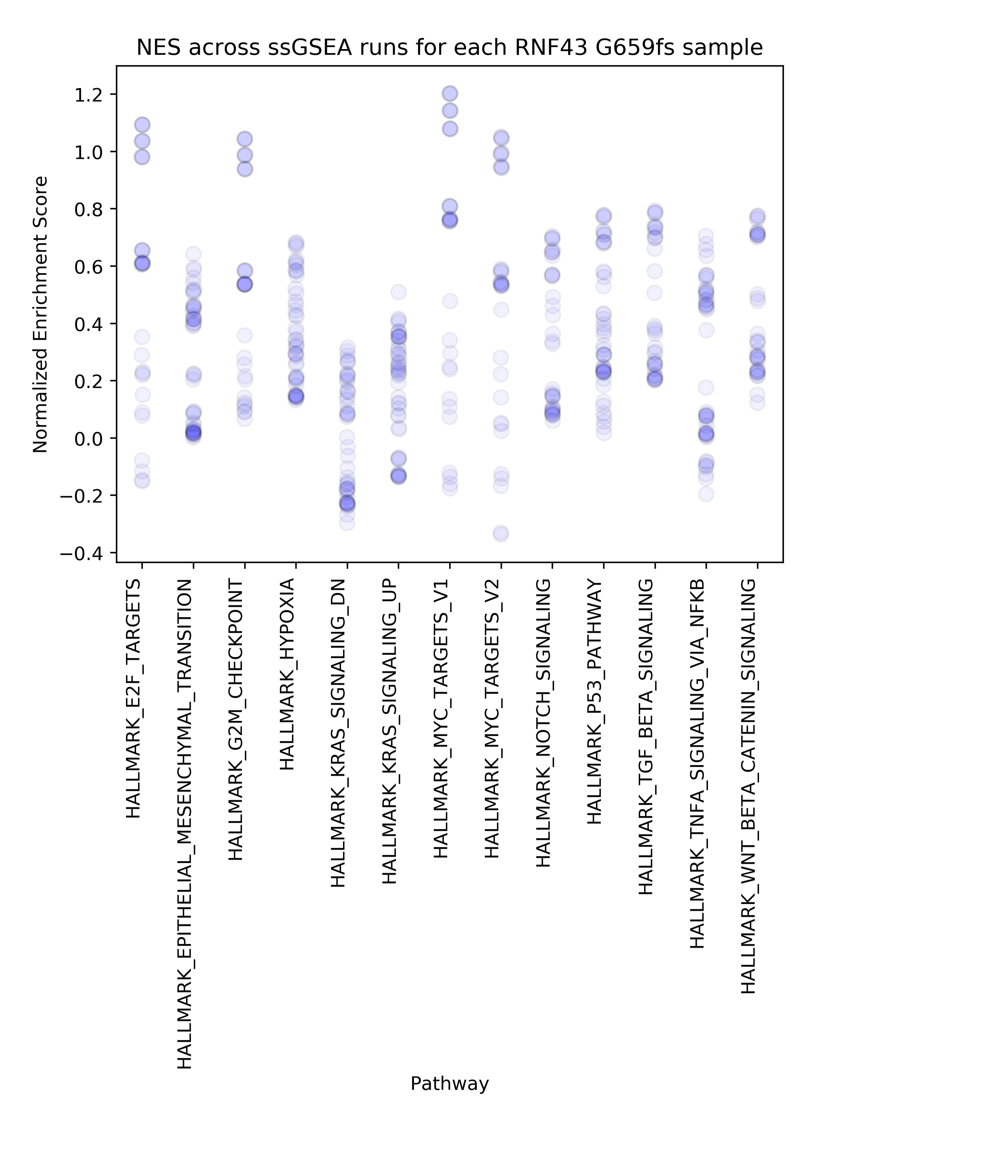

Supplement: S8 Fig — Distribution of normalized enrichment scores for each of the 12 variations of ssGSEA runs for each of the 4 RNF43 G659fs replicate across the validated Hallmark pathways. (TIFF) [file pcbi.1009132.s008.tiff]

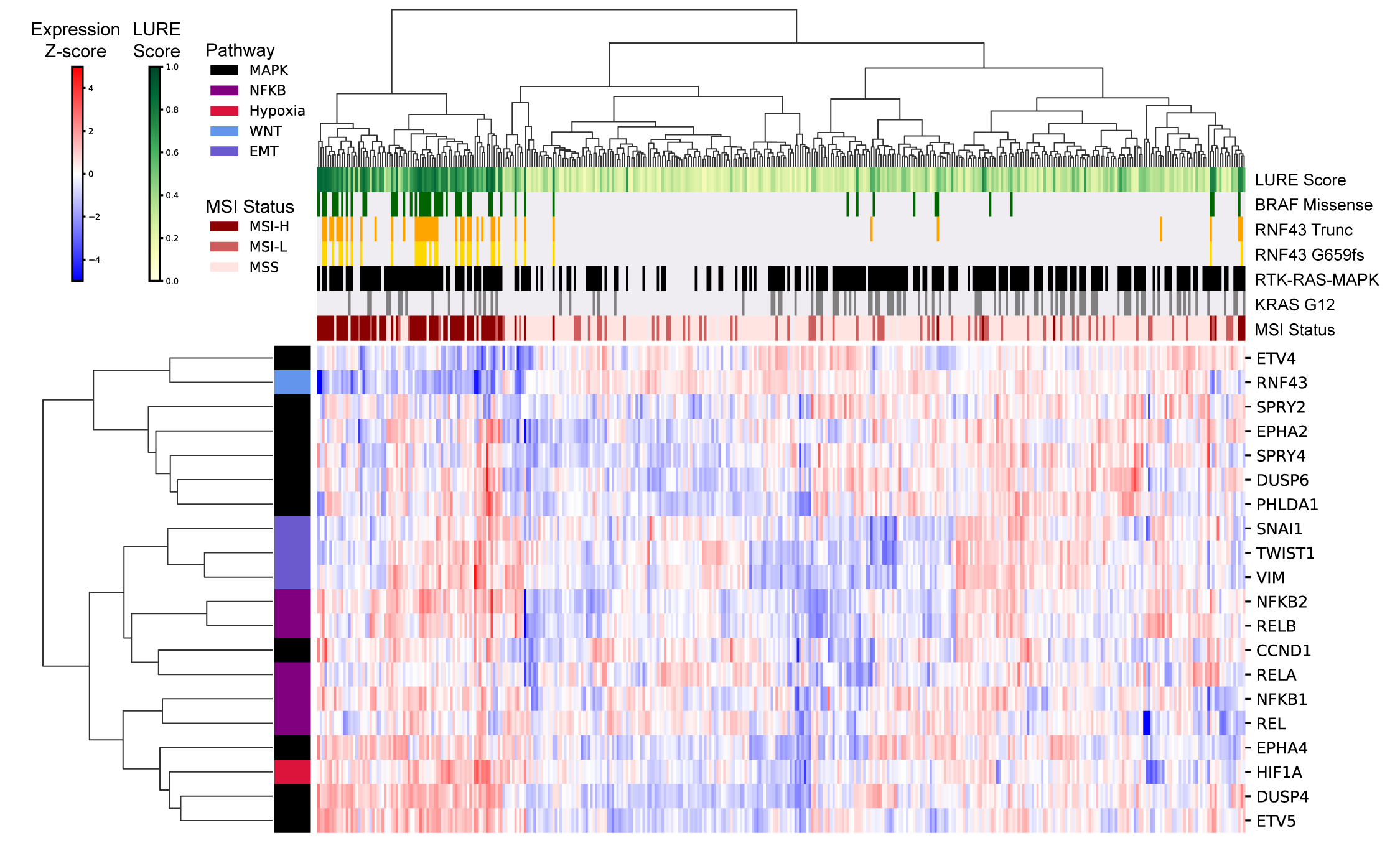

Supplement: S9 Fig — (A) Hierarchical clustering of gene expression (z-score) across TCGA COAD samples using pathway marker genes. The top color bar shows the LURE BRAF missense mutation score, BRAF missense status, RNF43 truncating status, RNF43 G659fs status, driver event status for genes in the RTK-RAS-MAPK pathway [37], KRAS G12(A,C,D,R,S or,V) status, and microsatellite instability status (high, low, or stable) status. The side color bar shows what pathway each gene is a marker for. (B,C) ETV5 and DUSP4 expression (log2(x+1) transformed RSEM normalized counts) across RNF43 and BRAF status. (Kruskal Wallis test across groups. ns: 5.00e-02 < p < = 1.00e+00, *: 1.00e-02 < p < = 5.00e-02, **: 1.00e-03 < p < = 1.00e-02, ***: 1.00e-04 < p < = 1.00e-03, ****: p < = 1.00e-04) (D) Subset of clustering from (A) showing only samples with RNF43 G659fs (and no other truncating RNF43 variants) with WT BRAF. (TIFF) [file pcbi.1009132.s009.tiff]
