# Supplementary material for: eVIP2: Expression-based variant impact phenotyping to predict the function of gene variants
Source: PLoS Comput Biol. 2021 Jul 2;17(7):e1009132. doi: 10.1371/journal.pcbi.1009132 (PMC8281988; doi:10.1371/journal.pcbi.1009132)
Supplement: S1 Table — (DOCX) [file pcbi.1009132.s010.docx]

**S1 Table. Cignal Reporter Assay used to measure pathway activity and their associated hallmark pathways.**

| **Tube number** | **Pathway** | **Cignal Reporter Assay** | **Hallmark Pathway** |
| --- | --- | --- | --- |
| 1 | Wnt | TCF/LEF Cignal Reporter Assay | HALLMARK_WNT_BETA_CATENIN_SIGNALING |
| 2 | Notch | RBP-Jκ Cignal Reporter Assay | HALLMARK_NOTCH_SIGNALING |
| 3 | p53/DNA Damage | p53 Cignal Reporter Assay | HALLMARK_P53_PATHWAY |
| 4 | TGFβ | SMAD2/3/4 Cignal Reporter Assay | HALLMARK_TGF_BETA_SIGNALING |
| 5 | Cell cycle/pRb-E2F | E2F/DP1NFκB Cignal Reporter Assay | HALLMARK_E2F_TARGETS,  HALLMARK_G2M_CHECKPOINT |
| 6 | NFκB | NFκB Cignal Reporter Assay | HALLMARK_TNFA_SIGNALING_VIA_NFKB |
| 7 | Myc/Max | Myc/Max Cignal Reporter Assay | HALLMARK_MYC_TARGETS_V1  HALLMARK_MYC_TARGETS_V2 |
| 8 | Hypoxia | HIF1A Cignal Reporter Assay | HALLMARK_HYPOXIA |
| 9 | MAPK/ERK | Elk-1/SRF Cignal Reporter Assay | HALLMARK_KRAS_SIGNALING_UP,  HALLMARK_KRAS_SIGNALING_DN |
| 10 | MAPK/JNK | AP-1 Cignal Reporter Assay | HALLMARK_KRAS_SIGNALING_UP,  HALLMARK_KRAS_SIGNALING_DN |
