# Supplementary material for: eVIP2: Expression-based variant impact phenotyping to predict the function of gene variants
Source: PLoS Comput Biol. 2021 Jul 2;17(7):e1009132. doi: 10.1371/journal.pcbi.1009132 (PMC8281988; doi:10.1371/journal.pcbi.1009132)
Supplement: S2 Table — Pathway reporter assay results are more consistent with eVIP Pathway prediction than with GSEA. Results from a pathway reporter array using different sample group comparisons (first 3 columns). Pathways were considered significant with p-value under .05, then the direction of difference is reported as inhibited or activated. The last two columns are eVIP Pathway and GSEA Investigate Gene Sets results (GOF = Gain of Function, COF = Change of function). GSEA Investigate Gene Sets was run on RNF43 G659fs mutation-specific genes. Only significant pathways (FDR q-value < .05) that contain at least 10 genes in the overlap are shown. (DOCX) [file pcbi.1009132.s011.docx]

**S2 Table. Reporter assay results compared with eVIP Pathways and GSEA**

|  | **WT relative to control** | **659fs relative to the control** | **659fs relative to WT** | **Westerns - 659fs relative to WT** | **eVIP Pathways** | **GSEA Investigate Gene Sets overlaps (mutation-specific genes)** |
| --- | --- | --- | --- | --- | --- | --- |
| **Wnt** | inhibited |  | activated |  |  |  |
| **Notch** |  |  |  |  |  |  |
| **p53** |  |  |  |  | GOF | significant overlap |
| **TGFBeta** |  |  |  |  |  |  |
| **Cell Cycle** | inhibited |  | activated |  |  |  |
| **NF-KB** |  | activated | activated | activated | COF | significant overlap |
| **Myc** |  |  |  |  |  |  |
| **Hypoxia** |  | activated | activated | activated | COF | significant overlap |
| **ERK** |  | activated | activated | activated | GOF | significant overlap |
| **JNK** |  | activated | activated | activated | GOF | significant overlap |

**S2 Table. Reporter assay results compared with eVIP Pathways and GSEA**

Pathway reporter assay results are more consistent with eVIP Pathway prediction than with GSEA. Results from a pathway reporter array using different sample group comparisons (first 3 columns). Pathways were considered significant with p-value under .05, then the direction of difference is reported as inhibited or activated. The last two columns are eVIP Pathway and GSEA Investigate Gene Sets results (GOF=Gain of Function, COF = Change of function). GSEA Investigate Gene Sets was run on RNF43 G659fs mutation-specific genes. Only significant pathways (FDR q-value <.05) that contain at least 10 genes in the overlap are shown.
